# An Updated Review of the Management of Chronic Heart Failure in Patients with Chronic Kidney Disease

**DOI:** 10.31083/j.rcm2504144

**Published:** 2024-04-11

**Authors:** Ella Tumelty, Isaac Chung, Sabba Hussain, Mahrukh Ayesha Ali, Harshavardhani Addada, Debasish Banerjee

**Affiliations:** ^1^Renal and Transplantation Unit, St George’s University Hospitals NHS Foundation Trust London, SW17 0QT London, UK; ^2^Cardiovascular and Genetics Research Institute St George’s University of London, SW17 0QT London, UK

**Keywords:** heart failure, chronic kidney disease, management, review

## Abstract

Chronic kidney disease (CKD) is common in patients with heart failure (HF) and is associated with high morbidity and mortality. There has been remarkable progress in the treatment of HF over recent years with the establishment of guideline-directed medical therapies including: (1) Beta-blockers, (2) renal angiotensin aldosterone system (RAAS) inhibition (i.e., angiotensin-converting enzyme inhibitor [ACEi], aldosterone receptor blocker [ARB] or angiotensin receptor-neprilysin inhibitor [ARNI]); (3) mineralocorticoid receptor antagonists (MRA), and (4) sodium-glucose cotransporter-2 inhibitors (SGLT2i). However, there are challenges to the implementation of these medications in patients with concomitant CKD due to increased vulnerability to common side-effects (including worsening renal function, hyperkalaemia, hypotension), and most of the pivotal trials which provide evidence of the efficacy of these medications excluded patients with severe CKD. Patients with CKD and HF often have regular healthcare encounters with multiple professionals and can receive conflicting guidance regarding their medication. Thus, despite being at higher risk of adverse cardiovascular events, patients who have both HF and CKD are more likely to be under-optimised on evidence-based therapies. This review is an updated summary of the evidence available for the management of HF (including reduced, mildly reduced and preserved left ventricular ejection fraction) in patients with various stages of CKD. The review covers the evidence for recommended medications, devices such as implantable cardioverter-defibrillator (ICD), cardiac resynchronization therapy (CRT), intravenous (IV) iron, and discusses how frailty affects the management of these patients. It also considers emerging evidence for the prevention of HF in the cohort of patients with CKD. It synthesises the available evidence regarding when to temporarily stop, continue or rechallenge medications in this cohort. Chronic HF in context of CKD remains a challenging scenario for clinicians to manage, which is usually complicated by frailty, multimorbidity and polypharmacy. Treatment should be tailored to a patients individual needs and management in specialised cardio-renal clinics with a multi-disciplinary team approach has been recommended. This review offers a concise summary on this expansive topic.

## 1. Introduction 

Heart failure (HF) is not one pathological entity, but a clinical syndrome constituting symptoms (e.g., dyspnoea, peripheral oedema and fatigue) and signs (e.g., pulmonary crepitations, raised jugular venous pressure), due to a structural or functional abnormality of the heart leading to inadequate cardiac output and/or elevated intracardiac pressures [1]. HF is common, affecting 64 million people worldwide, and its prevalence is increasing [2]. In the UK, more than one million people live with HF and approximately 200,000 new diagnoses are made annually [3]. The prognosis of HF has improved over recent years, however, it remains poor with 5-year mortality rates estimated at 43.3% [4].

Chronic kidney disease (CKD) is another chronic disease epidemic, the incidence and prevalence of which is increasing [5]. CKD is defined using reduced estimated glomerular filtration rate (eGFR) (<60 mL/min/1.73 $m^{2}$) and/or indicators of renal damage such as proteinuria [6].

**Fig. 1.**
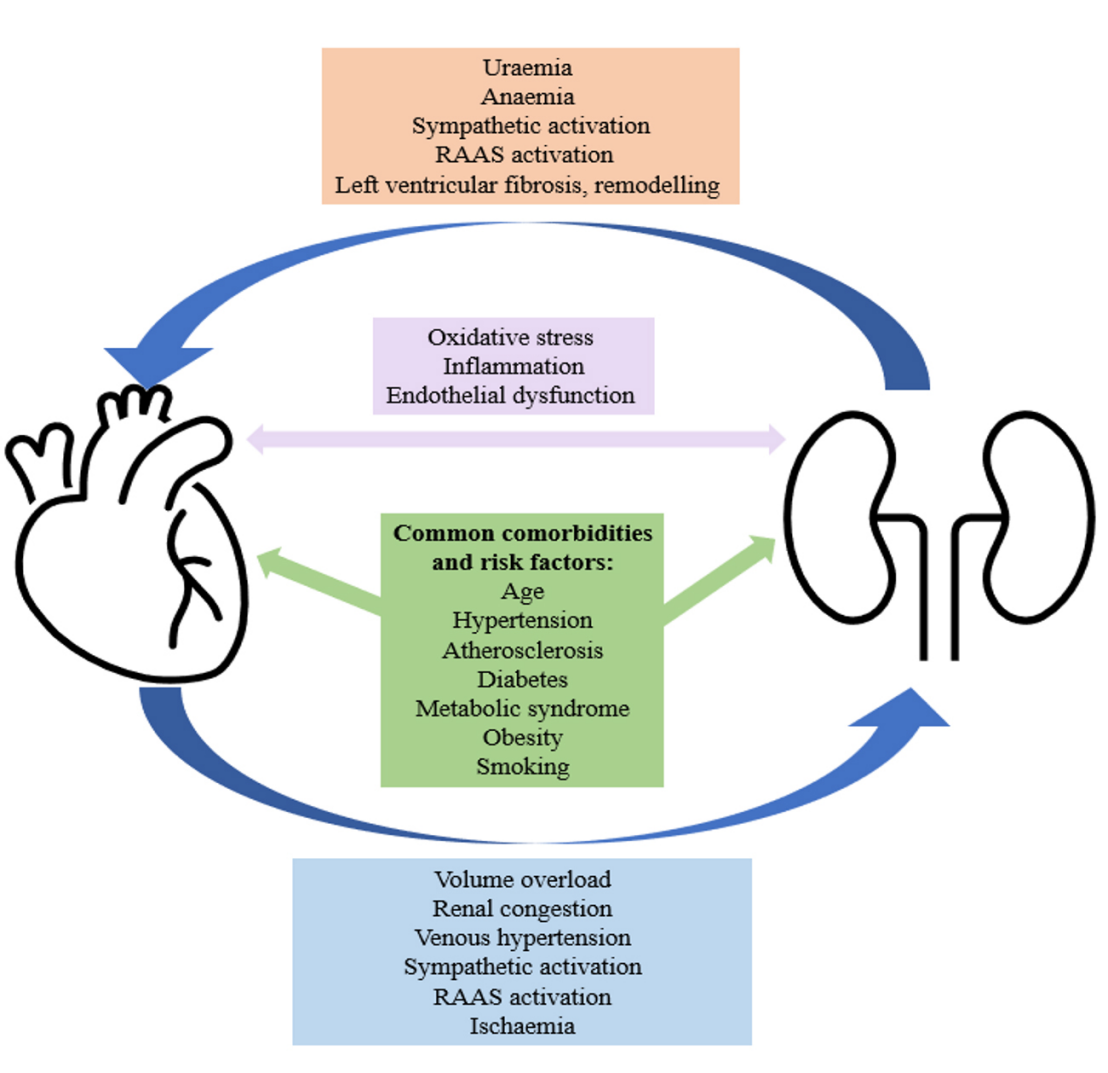
**A simplified diagram to demonstrate the complex and bidirectional relationship between CKD and HF**. CKD, chronic kidney disease; HF, heart failure; RAAS, renal angiotensin aldosterone system.

Nearly half of patients with HF have concomitant CKD [7]. There is a complex and bi-directional relationship between these two chronic conditions, with each increasing the risk of developing, and/or accelerating the progression of the other (Fig. 1) [8, 9]. In HF, volume overload can lead to renal congestion, venous hypertension, activation of the renal angiotensin aldosterone system (RAAS) and/or ischaemic damage to the kidneys. In CKD, the resultant anaemia and uraemia can lead to left ventricular fibrosis and remodelling. Furthermore, both conditions share several common comorbidities including hypertension, atherosclerosis, type 2 diabetes mellitus, obesity and metabolic syndrome, the prevalence of which are increasing [9, 10, 11].

CKD has consistently been found to carry the greatest population attributable risk for hospitalisation and all-cause mortality in patients with HF [7, 12, 13]. A meta-analysis found that all-cause mortality in HF patients with CKD was twice as high than for those without CKD (Odds Ratio [OR] 2.34, 95% confidence interval [CI] 2.20–2.50, *p* = 0.001) [7]. In the UK, whilst mortality rates for patients with HF have improved over the past 20 years, mortality rates remain static for patients with HF and CKD [14]. Renal impairment has been shown to predict HF mortality more accurately than left ventricular ejection fraction (LVEF) or New York Heart Association (NYHA) stage [15, 16], and CKD becomes more predictive for mortality as it progresses [14].

## 2. Categories of HF and CKD 

### 2.1 Left Ventricular Ejection Fraction (LVEF)

HF is primarily classified according to LVEF; reduced ≤40% (HFrEF), mildly reduced 41–49% (HFmrEF), and preserved ≥50% (HFpEF) [1]. HFrEF is well characterised, and the majority of historical trials to investigate the treatment of HF have been conducted in this subgroup. HFpEF (patients with signs and symptoms of HF with evidence of cardiac abnormalities, usually with increased natriuretic peptide levels, but with a ‘normal LVEF’) has been described for several years, however previous LVEF definitions have varied from >40%, >45%, ≥45%, >50%, or ≥50% [1]. This inconsistency led to the introduction of a relatively new category, HFmrEF, by the European Society of Cardiology (ESC) guidelines in 2016.

Several distinguishable features have been observed regarding each subgroup; patients with HFrEF are more likely to have ischaemic heart disease and are more likely to die or be hospitalised from a primary cardiovascular cause [17]. Patients with HFpEF are more likely to be older, female, more comorbid, and are more likely to die or be hospitalised from a non-cardiovascular cause [17]. HFpEF is more likely to be associated with hypertension, than ischaemia. Most analyses conclude that HFmrEF is more similar to HFrEF, however it shares some characteristics with HFpEF. Patients with HFmrEF have an increased prevalence of ischaemic heart disease like HFrEF, but other features are more comparable to HFpEF (lower cardiovascular risk, more likely to be hypertensive etc.) [17]. Evidence-based therapies for the management of HFrEF are well established. Comparatively, HFpEF and HFmrEF are areas of paucity of evidence. Until recently, there was no evidence for the management of HFpEF, but trials published in 2021 and 2022 respectively [18, 19], have now seen the introduction of the first evidence-based therapy for this cohort (discussed further in the SGLT2i section). Most evidence for HFmrEF is derived from subgroup analyses of randomised controlled trials (RCT’s) which were not intentionally designed to investigate this cohort, but included some patients with LVEF 41–50% [1]. There are limitations to this classification system, not least due to the variability in performance and interpretation of echocardiograms, but also because LVEF measurements can change over time. Furthermore, this system is a blunt instrument to categorise HF patients who likely, especially in HFmrEF and HFpEF, represent considerable phenotypic heterogeneity.

### 2.2 New York Heart Association (NYHA) Classification 

The NYHA Classification tool is a simple way to categorise HF patients based on their functional abilities, which has been widely used for over 100 years. It categorises patient from class one (no symptoms) to class four (severe symptoms), (Table 1, Ref. [20]). Its relevance and reliability in predicting outcomes has been deliberated, but it remains ubiquitous within HF literature, and as such, we have considered the representation of each of the NYHA classes in HF RCT’s in this review [21].

**Table 1.** **NYHA Classification**.

NYHA Classification [20]	Description
Class I	No limitation of physical activity. Ordinary physical activity does not cause undue fatigue, palpitation or dyspnoea.
Class II	Slight limitation of physical activity. Comfortable at rest but ordinary physical activity results in fatigue, palpitation or dyspnoea.
Class III	Marked limitation of physical activity. Comfortable at rest but less than ordinary physical activity results in fatigue, palpitation or dyspnoea.
Class IV	Unable to carry out any physical activity without discomfort. Symptoms at rest. If any physical activity is undertaken, discomfort is increased.

NYHA, New York Heart Association. Adapted from *Dolgin M, Association NYH, Fox AC, Gorlin R, Levin RI, New York Heart Association. Criteria Committee. Nomenclature and criteria for diagnosis of diseases of the heart and great vessels. 9th ed. Boston, MA: Lippincott Williams and Wilkins; March 1, 1994 [20]*.

### 2.3 CKD Stages 

As per the Kidney Disease Improving Global Outcomes (KDIGO) 2012 guidelines, patients with CKD should be categorised into stages G1-5 based on eGFR (mL/min/1.73 $m^{2}$), as well as A1–A3 based on extent of albuminuria (mg/mmol) (Table 2, Ref. [22]).

**Table 2.** **Adopted from KIDGO 2012 Clinical Practice Guideline for the Evaluation and Management of Chronic Kidney Disease [22]**.

			Persistent albuminuria categories		
			A1	A2	A3
			<30 mg/g	30–300 mg/g	>300 mg/g
			<3 mg/mmol	3–30 mg/mmol	>30 mg/mmol
eGFR categories (mL/min/1.73 $m^{2}$)	G1	≥90			
	G2	60–89			
	G3a	45–59			
	G3b	30–44			
	G4	15–29			
	G5	<15			

**Colour key:** Green = low risk (if no other markers of kidney disease, no CKD). Yellow = moderately increased risk. Orange = High risk. Red = Very high risk. KDIGO, kidney disease improving global outcomes; CKD, chronic kidney disease; eGFR, estimated glomerular filtration rate.

### 2.4 Challenges within This Population 

The prognosis of HFrEF has improved considerably since the introduction of evidence-based medical therapies. The most recent guidelines for HFrEF advocate a ‘quadruple therapy’ approach using the following medications: (1) Beta-blockers, (2) RAAS inhibition (i.e., angiotensin-converting enzyme inhibitor [ACEi], aldosterone receptor blocker [ARB], or angiotensin receptor-neprilysin inhibitor [ARNI]); (3) mineralocorticoid receptor antagonists (MRA) and (4) sodium-glucose cotransporter-2 inhibitors (SGLT2i’s) [1].

However, there is concern regarding the use of these medications in patients with CKD, due to the often associated rise in creatinine [23] and potassium [8], greater risk of hypotension [24] and the fact that patients with severe renal dysfunction were excluded from the pivotal RCT’s, so there is limited evidence of their efficacy within this population (Table 3, Ref. [18, 19, 25, 26, 27, 28, 29, 30, 31]). These patients often have multiple healthcare encounters e.g., with nephrologists, cardiologists, general practitioners, internal medicine physicians, and may receive conflicting advice regarding these medications. Thus, despite being at higher risk of adverse cardiovascular events, patients who have both HF and CKD are less likely to be optimised on guideline-directed medical therapy for HF [32].

**Table 3.** **Summary of pivotal trials providing evidence for HF: management, in those with and without chronic kidney disease**.

Trial	Exclusion	<60 mL/min/1.73 $m^{2}$	>60 mL/min/1.73 $m^{2}$
DAPA-HF [25]	eGFR <30	0.72 [0.66–0.86]	0.76 [0.63–0.92]
DELIVER [19]	eGFR <25	0.81 [0.69–0.94]	0.84 [0.70–1.00]
EMPEROR-Preserved [18]	eGFR <20	0.78 [0.66–0.91]	0.81 [0.66–1.00]
EMPEROR-Reduced [26]	eGFR <20	0.83 [0.69–1.00]	0.67 [0.55–0.83]
SOLOIST-HF [27]	eGFR <30	0.59 [0.44–0.79]	0.90 [0.58–1.37]
PIONEER-HF [28]	eGFR <30	0.73 [0.61–0.87]	0.70 [0.59–0.84]
PARAGON-HF [29]	eGFR <30	0.79 [0.66–0.95]	1.01 [0.80–1.27]
PARADIGM-HF [30]	eGFR <30	similar	similar
EMPHASIS [31]	eGFR <30	similar	similar

eGFR, estimated glomerular filtration rate; HF, heart failure.

This review will discuss the existing evidence for managing chronic HF (HFrEF, HFmrEF, HFpEF) in patients with various stages of CKD.

## 3. Diuretics

Diuretics are indicated to clinically improve congestion in HF (i.e., extracellular fluid, peripheral oedema), and they should be used to achieve euvolemia using the lowest required dose [33]. Diuretics increase the excretion of sodium and water in urine (natriuresis and diuresis), with the various subtypes achieving this through different areas of the nephron e.g., loop-diuretics (such as furosemide) act on the ascending loop of Henle, whereas thiazide-like diuretics (e.g., indapamide) act on the early distal convoluted tubule [34, 35]. There is no evidence for diuretics improving outcomes in HF, hence, their requirement in chronic HF should be re-assessed regularly, and the dose reduced, if possible, to allow up titration of medical therapies with prognostic benefit [36]. However, diuretics are recommended for improving symptoms across all HF subtypes (HFrEF, HFmrEF and HFpEF) [37].

There are specific challenges with the use of diuretics in patients with HF and CKD. Many patients with CKD have renal sodium affinity, leading to diuretic resistance [38]. There are several mechanisms which may explain this, including albuminuria and hypoproteinaemia, leading to an increased volume of distribution of the diuretic and reduced delivery to the kidney [39].

### 3.1 Diuretics in Acute HF 

This review primarily focuses on the management of chronic HF. However, there are a few important points and recent updates regarding the use of diuretics in acute HF which we would like to highlight.

In acute HF, the parenteral administration of diuretics is preferable, as this has a higher bioavailability than oral and bypasses gastrointestinal oedema resulting in quicker absorption [40]. Studies have found no different in efficacy between loop diuretics infused continuously or as twice-daily boluses, but a once-daily bolus regimen should be avoided [41].

Diuretics, especially with high doses, can transiently impact renal function, cause imbalances in electrolytes (including hyponatraemia and hypokalaemia), and lead to hypovolaemia [42]. During the management of acute HF, any diuretic-associated increase in creatinine should be evaluated within the context of any change in clinical status. A diuretic-associated increase in creatinine which is associated with signs of decongestion may represent effective diuresis [43], and as shown in the Diuretic Optimization Strategies Evaluation (DOSE) study, worsening renal function in this context can paradoxically be a positive prognostic indicator [44]. However, a rising creatinine with no improvement in signs of congestion is a poor prognostic marker [38].

ESC guidelines recommend monitoring a patient’s diuretic response using either spot urinary sodium concentration two or six hours post diuretic dose or hourly urine output and amending the diuretic regime accordingly [1]. Previous trials have investigated various methods of improving diuretic response in acute HF [45, 46, 47, 48]. For example, to overcome the resistance caused by hypoalbuminaemia, trials have investigated the utility of delivering furosemide alongside albumin to improve diuresis, however, no effect was observed [45, 46].

Furthermore, the 2023 ESC guidelines update highlighted two recent clinical trials investigating a dual-diuretic approach for acute HF – the ADVOR trial (Acetazolamide in Acute Decompensated Heart Failure with Volume Overload) [47] and the CLOROTIC trial (Combining loop with thiazide diuretics for decompensated heart failure) [48]. The ADVOR trial randomised 519 patients with acute HF with a median eGFR of 38 mL/min/1.73 $m^{2}$ to either 500 mg IV acetazolamide or placebo, in addition to standard IV loop diuretic treatment. ADVOR demonstrated increased rates of successful decongestion in the acetazolamide arm (Relative risk, RR 1.48; 95% CI 1.17–1.82, *p* < 0.001), with similar rates of electrolyte abnormalities and adverse events across both arms [47].

The CLOROTIC trial investigated the addition of oral hydrochlorothiazide to standard IV furosemide in 230 patients with acute HF, with median eGFR 43 mL/min/1.73 $m^{2}$ [48]. Weight loss was significantly greater in those randomised to hydrochlorothiazide compared to placebo, at 72 hours (–2.3 vs –1.5 kg, *p* = 0.002) and 96 hours (–2.5 kg vs –1.5 kg, *p* < 0.001). Worsening renal function (defined as reduction of eGFR of >50% or increase in creatinine >26.5 µmol/L) was more common in those who received hydrochlorothiazide (46.5%), than placebo (17.2%), *p* < 0.001. There was no difference in dyspnoea scores, hypokalaemia, mortality or hospitalisations.

Regarding both trials, ESC concluded that further safety and outcome data was required prior to either of the dual-diuretic strategies being implemented into guidelines.

### 3.2 Diuretics in Chronic HF 

Generally, concomitant use of various classes of diuretics may be necessary for patients with CKD and HF with diuretic resistance. Thiazide diuretics are less effective in advanced CKD (due to earlier absorption of sodium, reducing the efficacy of thiazide diuretics impact) [49]. Often, loop diuretics and metolazone are used simultaneously [50]. Importantly, medications such as MRA’s, SGLT2i’s and ARNI’s also have some diuretic effect. Practically, patients with CKD should be treated with loop diuretics to achieve euvolemia if indicated. Serum biomarkers (including creatinine and potassium) and the patient’s fluid status should be monitored closely [10].

## 4. Renin-Angiotensin Aldosterone System (RAAS) Inhibition 

### 4.1 ACEi and ARB

#### 4.1.1 ACEi/ARB in HFrEF 

There has been consistent RCT and meta-analysis evidence over the past 30 years demonstrating the benefits of ACEi’s in HFrEF, and subsequently ACEi’s have formed the cornerstone of HFrEF management [51, 52, 53, 54, 55, 56, 57]. The benefits demonstrated have included improved LVEF [51], reduced mortality [52, 53, 54, 56, 58, 59] and reduced hospitalization [53, 54]. The survival benefit has been demonstrated in mild, moderate and severe HF [53, 58, 60, 61].

However, the cited studies all excluded patients with severe CKD, and had a median baseline creatinine exclusion cut-off of 221 µmol/L (Interquartile range [IQR] 21) (Table 4, Ref. [51, 52, 53, 54, 55, 56, 57, 58, 59, 60, 61, 62, 63, 64]). Subgroup analyses of CKD patients included in these trials show no outcome modification by renal function at baseline, however, still included very few, if any patients with severe CKD [65, 66]. Thus, there is evidence that the benefit of ACEi is consistent in patients with mild-moderate CKD [65, 66]. There is only inconsistent and moderate evidence of benefit in patients with CKD stage G4, however, there is also no suggestion of harm [67]. Further evidence is warranted.

**Table 4.** **Summary of pivotal RCT’s for use of ACEi’s for management of HF**.

Trial name, year (Ref)	N	Main outcome	Intervention *(target dose) *vs comparator *(target dose)*	LVEF inclusion criteria	Renal exclusion criteria	NYHA class of participants	Overall results (Primary outcome) (95% CI; *p* value)
Captopril, 1983 [51]	92	(1) Change in NYHA class	Captopril* (50 mg TDS) *vs placebo	Not stated. Mean baseline 19%	Creatinine clearance ≥50 mL/min	II – 40.2%	NYHA Class (adjusted change): Captopril –0.52, Placebo –0.03; *p* = 0.0004
		(2) Change in exercise tolerance				III – 56.5%	Exercise Tolerance (adjusted % change): Captopril 24.3%, Placebo 0.4%; *p* = 0.007
		(3) Change in LVEF				IV – 3.3%	EF (% change): Captopril 16.2%, Placebo –1.8; *p * < 0.05
CONSENSUS, 1987 [58]	253	All-cause mortality at 6 months	Enalapril *(5 mg–20 mg BD) *vs placebo	Not stated	Creatinine >300 µmol/L	IV – 100%	Enalapril 33 (26%), Placebo 55 (44%), risk reduction 40%; *p* = 0.002
SAVE, 1992 [52]	2231	All-cause mortality	Captopril* (25–50 mg TDS)* vs placebo	<40%	Creatinine >221 µmol/L (2.5 mg/dL)	Not stated	Captopril 228 (20%), placebo 275 (25%), risk reduction 19% (95% CI 3 to 32%; *p* = 0.019)
SOLVD-T, 1991 [53]	2569	(1) All-cause mortality	Enalapril* (2.5 mg–10 mg BD) *vs placebo	≤35%	Creatinine >221 µmol/L (2.5 mg/dL) or on dialysis	I – 10.9%	All-cause mortality: Enalapril 452 (35.2%), Placebo 510 (39.7%), risk reduction 16% (95% CI 5 to 26%; *p* = 0.0036)
		(2) Composite outcome: HF hospitalisation or mortality				II – 56.7%	HF Hospitalisation + mortality: Enalapril 613 (23.9%), Placebo 736 (28.6%), risk reduction 26% (95% CI 18 to 34%; *p * < 0.0001)
						III – 30.4%	
						IV – 1.7%	
SOLVD-P, 1992 [54]	4228	(1) All-cause mortality	Enalapril* (2.5 mg–10 mg BD) *vs placebo	≤35%	Creatinine >221 µmol/L (2.5 mg/dL) or on dialysis	I – 66.7%	All-cause mortality: Enalapril 313 (7.4%), placebo 334 (7.9%), risk reduction 8% (95 % CI –8% to 21%; *p* = 0.30)
		(2) Composite outcome: Development symptomatic HF or mortality				II – 33.0%	Symptomatic HF + mortality: Enalapril 630 (14.9%), placebo 818 (19.3%), risk reduction 29% (95% CI 21 to 36%; *p * < 0001)
		(3) Composite outcome: Hospitalisation for HF or mortality					HF Hospitalisation + mortality: Enalapril 434 (10.3%), placebo 518 (12.3%), risk reduction 20% (95% CI 9 to 30%; *p * < 0.001)
AIRE, 1993 [63]	2006	All-cause mortality	Ramipril *(2.5–5 mg BD)* vs placebo	Not stated	Not stated - states 289 excluded due to “renal failure”	II/III – 100%	All-cause mortality: Ramipril 170 (17%), Placebo 222 (23%), Risk reduction 27% (95 % CI 11% to 40%; *p* = 0.002)
DIG enalapril, 1991 [55]	145	(1) Functional capacity	Enalapril *(20 mg BD)* vs digoxin *(dose based on body weight, initial dose from 0.125–0.375 mg*)	<50%	Creatinine >130 µmol/L (1.5 mg/dL)	II/III – 100%	(1) Functional capacity: Week 4:* Improvement *- enalapril 13 (18%), digoxin 7 (10%). *No change –* Enalapril 55 (76%), Digoxin 49 (67%). *Worsening-* enalapril 4 (6%), digoxin 17 (23%) (Chi-square =13.98, df = 2, *p* = 0.001)
		(2) Exercise time		Not stated. Mean baseline 30%			Week 14: *Improvement -* enalapril 13 (18%), digoxin 14 (19%). *No change* – Enalapril 50 (69%), Digoxin 37 (51%). *Worsening-* enalapril 9 (13%), digoxin 22 (30%) (Chi-square = 7.32, df = 2, *p* = 0.026)
		(3) Change in echocardiographic dimensions					(2) Exercise time: Significant improvement in each group, no difference between groups (*p* = 0.497)
							(3) ECHO features: Improvement in both, no difference between groups
TRACE, 1995 [59]	1749	All-cause mortality	Trandolapril *(2 mg OD)* vs placebo	<35%	Creatinine ≥200 µmol/L (2.3 mg/dL)	1–41%	All-cause mortality at 4 years: Trandolapril 304 (34.7%) vs Placebo 369 (42.3%), relative risk 0.78 (95% CI 0.67 to 0.91; *p* = 0.001)
						Others not specified	
V-HeFT II, 1991 [56]	804	Peak oxygen consumption during exercise (mL/kg/min)	Enalapril *(20 mg OD)* vs HID: [Hydralazine *(300 mg OD) *+ ISDN *(160 mg OD)*]	<45%	Not stated	I – 5.7%	Peak oxygen consumption during exercise (mL/kg/min): Enalapril 0.2 vs HID 0.8 (*p* = 0.02)
		Change in LVEF (%)				II – 51.0%	LVEF increase: Enalapril 0.021 vs HID 0.033 (*p* = 0.026)
		Mortality at 2 years				III – 42.9%	Cumulative 48m mortality: Enalapril 0.18 vs HID 0.25 (*p* = 0.016)
						IV – 0.4%	
NETWORK, 1998 [60]	1532	Composite of death, HF related hospitalisation or worsening HF	Enalapril *(2.5 mg BD)* vs Enalapril *(5 mg BD)* vs Enalapril *(10 mg BD)*	None	Creatinine >200 µmol/L	II – 65%	Composite outcome: Enalapril 2.5 mg BD – 62 (12.3%), Enalapril 5 mg BD – 66 (12.9%), Enalapril 10 mg BD – 76 (14.7%) – non-significant
						III – 33%	
						IV – 2%	
ATLAS, 1999 [61]	3164	(1) All-cause mortality	Low dose lisinopril *(2.5–5.0 mg OD)* vs High dose Lisinopril *(32.5–25 mg OD)*	≤30%	Creatinine >221 µmol/L (2.5 mg/dL)	II – 15.6%	All-cause mortality: 8% lower in high-dose group (*p* = 0.128)
		(2) Composite outcome: death or hospitalisation for any reason				III – 77.3%	Death + hospitalisation for any cause: 12% lower risk in high-dose group (*p* = 0.002)
						IV – 7.1%	
Munich Mild HF Trial – MHFT, 1993 [57]	170	(1) Progression of HF to NYHA IV	Captopril *(25 mg BD)* vs Placebo	Not stated. Mean baseline 34.8%	Renal artery stenosis/renal failure requiring dialysis	I – 30.6%	Progression of HF: Tx 9 patients (10.8%), vs placebo 23 patients (26.4%), *p* = 0.01
		(2) Death due to HF				II – 59%	Death due to HF: Tx 4 patients (4.8%), vs placebo 11 patients (12.6%), *p* value 0.104
						III – 27.6%	
FEST, 1995 [64]	308	Maximal bicycle exercise time	Fosinopril *(40 mg OD)* vs Placebo	≤35%	Significant renal dysfunction	II – 64.6%	Median change from baseline (seconds) – fosinopril 40, placebo 24, *p* = 0.029
						III – 35.4%	
PEP-CHF, 2006 [62]	850	Composite of all-cause mortality or unplanned HF related hospital admission.	Perindopril *(4 mg OD) *vs Placebo	Equivalent to ≥40% (Wall motion index of <1.4)	Creatinine >200 µmol/L	I/II – 75.8%	Perindopril – 100, Placebo – 107 (HR 0.919: 95% CI 0.700–1.208; *p* = 0.545)
						III/IV – 24.2%	

Abbreviations used in Table 4: AIRE, acute infarction ramipril efficacy; ATLAS, assessment of treatment with lisinopril and survival; BD, twice a day; CI, confidence interval; CONSENSUS, effects of enalapril on mortality in severe congestive heart failure; dL, decilitre; ECHO, echocardiogram; FEST, fosinopril efficacy/safety trial; HF, heart failure; HID, hydralazine and isosorbide dinitrate; HR, hazard Ratio; ISDN, isosorbide dinitrate; LVEF, left ventricular ejection fraction; mg, milligram; min, minute; mL, millilitre; NYHA, New York Heart Association Classification; OD, once a day; PEP-CHF, perindopril for elderly people with chronic heart failure; RCT, randomised controlled trial; ACEi, angiotensin-converting enzyme inhibitor; TDS, three times per day; EF, ejection fraction.

The evidence for ARB’s in HFrEF is more inconsistent than that for ACEi’s, but there is evidence for their use, particularly in reducing hospital admissions and where ACEi’s are not tolerated (Table 5, Ref. [68, 69, 70, 71, 72, 73, 74, 75, 76, 77, 78]) [79]. The Evaluation of Losartan in the Elderly Study, Elite I and the Losartan Heart Failure Survival Study, Elite II (ELITE) studies compared losartan to captopril and found no significant difference in mortality or worsening renal function, but that losartan was significantly better tolerated than captopril [68, 69]. The ESC guidelines recommend ARB’s are used in patients unable to tolerate an ACEi/ARNI [1]. These trials also excluded patients with severe renal impairment (Table 5). However in a post-hoc analysis of the ValHeFT trial, even at severe CKD levels (eGFR 30), the treatment effect in favour of valsartan was still observed [70]. Similarly to ACEi’s, there is strong evidence for CKD stages G1-3, but further evidence is needed in patients with CKD stages G4/5 CKD, and subsequently patients should be monitored carefully, and dose modification may be necessary [50].

**Table 5.** **Summary of pivotal RCT’s for use of ARB for management of HF**.

Trial name, year (Ref)	N	Main outcome	Intervention *(target dose) *vs comparator *(target dose)*	LVEF inclusion criteria	Renal exclusion criteria	NYHA class of participants	Overall results (Primary outcome) (95% CI; *p* value)
ELITE, 1997 [68]	722	Persisting increase in serum creatinine ≥26.5 µmol/L	Losartan *(50 mg OD)* vs captopril *(50 mg TDS)*	≤40%	Creatinine ≥221 µmol/L (2.5 mg/dL)	II – 64.8%	HR 0.98 (95% CI 0.49–1.36; *p* = 0.63)
						III – 33.5%	
						IV – 1.7%	
ELITE-II, 2000 [69]	3152	All-cause mortality	Losartan *(50 mg OD) *vs captopril *(50 mg TDS)*	≤40%	Creatinine >221 µmol/L (2.5 mg/dL)	II – 51.9%	Losartan 280 (17.7%) vs captopril 250 (15.9%)
						III – 43.5%	HR 1.13 (95.7% CI 0.95–1.35, *p* = 0.16)
						IV – 4.6%	
CHARM Added/Alternative, 2003 [73, 74, 75, 76]	4576	Composite of CVS death or HF hospitalisation	Candesartan *(32 mg OD)* vs placebo	≤40%	Creatinine ≥265 µmol/L (>3 mg/dL)	II – 34.5%	Candesartan 817 (35.7%) vs placebo 944 (41.3%)
						III – 63.2%	HR 0.82 (95% CI 0.74–0.90, *p * < 0.001)
						IV – 3.3%	
CHARM-PRESERVE, 2003 [71, 73]	3023	Composite of CVS death or HF admission	Candesartan *(32 mg OD)* vs placebo	>40%	Creatinine ≥265 µmol/L (>3 mg/dL)	II – 61.0%	Candesartan 333 (22%), placebo 366 (24%), HR 0.89 (95% CI 0.77–1.03; *p* = 0.118); covariate adjusted 0.86 (95% CI 0.74–1.0; *p* = 0.051)
						III – 38.0%	
						IV – 2.0%	
HEAAL, 2009 [77]	3846	Composite of death or HF admission	Losartan* (150 mg OD) *vs losartan *(50 mg OD)*	≤40%	Creatinine >220 µmol/L	II – 69.3%	Grp 1 - 828 (43%) vs Grp 2 889 (46%)
						III – 30.0%	HR 0.90 (95% CI 0.82–0.99, *p* = 0.027)
						IV – 0.6%	
ValHeFT, 2001 [70, 78]	5010	(1) All-cause mortality	Valsartan *(160 mg BD)* vs placebo	<40%	Creatinine >221 µmol/L (2.5 mg/dL)	II – 61.8%	(1) All-cause mortality: Valsartan 495 (19.7%), placebo 484 (19.4%), RR 1.02 (98% CI 0.88–1.18, *p* = 0.80)
		(2) Composite of mortality and morbidity*				III – 36.2%	(2) Composite outcome: Valsartan 723 (28.8%), Placebo 801 (32.1%), RR 0.87 (97.5% CI 0.77–0.97, *p* = 0.009)
						IV – 1.9%	
I-PRESERVE, 2008 [72]	4218	Composite of all-cause mortality or CVS hospitalisation**	Irbesartan *(300 mg OD)* vs placebo	≥45%	Creatinine >221 µmol/L (2.5 mg/dL)	II – 21.1%	36% vs 37%; HR 0.95 (95% CI 0.86–1.05; *p* = 0.35)
						III – 76.2%	
						IV – 2.7%	

* Morbidity defined as cardiac arrest with resuscitation, HF hospitalisation or an episode of requiring IV vasodilator or inotropic therapy for a minimum four hours. ** Including HF, Myocardial infarction, unstable angina, arrhythmia, stroke. Abbreviations used in Table 5: ARB, angiotensin receptor blocker; BD, twice a day; CHARM, candesartan in heart failure assessment of reduction in mortality and morbidity; CI, confidence interval; CVS, cardiovascular; dL, decilitre; ELITE II, losartan heart failure survival study; Grp, group; HEAAL, effects of high-dose versus low-dose losartan on clinical outcomes in patients with heart failure; HF, heart failure; HR, hazard Ratio; I-PRESERVE, irbesartan in heart failure and preserved ejection fraction; LVEF, left ventricular ejection fraction; mg, milligram; NYHA, New York Heart Association Classification; OD, once a day; RCT, randomised controlled trial; Tx, treatment; ValHeFT, valsartan heart failure trial; µmol, micromol.

#### 4.1.2 ACEi/ARB in HFmrEF 

The ESC recommend that ACEi/ARB’s may be considered in patients with HFmrEF [1]. There are no specific interventional trials investigating the utility of ACEi/ARB’s for the management of HFmrEF. However, some implications (Level C evidence) can be drawn from observational data [17], as well as post-hoc analysis of RCT’s such as CHARM-Preserved and Irbesartan in Heart Failure and Preserved Ejection Fraction (I-PRESERVE) which included patients with LVEF >40% and >45% respectively [71, 72].

A post-hoc analysis of the CHARM trials demonstrated a reduction in hospitalisation rates for patients with HFmrEF treated with candesartan, compared to those on placebo (Hazard ratio, HR 0.76; 95% CI 0.61–0.96*; p = *0.02), which was similar to the reduction seen in HFrEF [80].

An analysis of ‘real-world’ large registry data found that many patients with HFmrEF are established on RAASi [17]. This may be because RAAS is indicated for other common comorbidities such as hypertension or diabetes, or that the patients previously had an LVEF of ≤40% which has improved following medical therapy and have continued on medical therapy, as is recommended in view of the Therapy withdrawal in REcovered Dilated cardiomyopathy (TRED)-HF trial results [81].

#### 4.1.3 ACE/ARB in HFpEF 

To date, there is no evidence based rationale for the use of ACEi/ARB for the management of HFpEF, including in those with CKD [8]. There have been several RCTs to investigate the potential of ACEi/ARB in HFpEF (The Perindopril in elderly people with chronic heart failure study [PEP-CHF] [62], Irbesartan in Patients with Heart Failure and Preserved Ejection Fraction [I-PRESERVE] [72], Effects of candesartan in patients with chronic heart failure and preserved left-ventricular ejection fraction [CHARM-Preserved]) [71] but none have met their primary endpoints. However, similarly to patients with HFmrEF, many patients with HFpEF are established on RAASi (>86% in the Prospective Comparison of ARNI with ARB Global Outcomes in HF with Preserved Ejection Fraction (PARAGON-HF) trial were taking ACEi/ARB at baseline) [1, 29].

#### 4.1.4 ACEi/ARB and Worsening Renal Function 

ACEi’s and ARB’s both cause vasodilatation of the efferent arteriole, leading to a reduction in nephron filtration pressure. This often leads to an increase in creatinine and reduction in eGFR when these medications are commenced or up titrated, which has caused hesitancy to commence these medications in patients with renal impairment. However, a post-hoc analysis of 6245 patients in the Studies of Left Ventricular Dysfunction (SOLVD) trials revealed that all-cause mortality, cardiovascular death and HF hospitalisation, were lower in those on ACEi’s, with no effect modification of declining eGFR [82]. In fact, in one analysis where the eGFR decline was presumed to be driven purely by the medication, a decline in eGFR of 10% at 2 weeks was significantly associated with reduced risk of death (HR = 0.87; 95% CI 0.77–0.99) and a decline of 35% at 2 weeks was significantly associated with reduced HF hospitalisations (HR 0.78; 95% CI 0.61–0.98) [82]. The Renin–Angiotensin System Inhibition in Advanced Chronic Kidney Disease (STOP-ACEi) trial provides further evidence to support the use of RAASi in patients with impaired renal function [83]. This trial of 411 patients with a median baseline eGFR of 18 mL/min/1.73 $m^{2}$ found that at three years, there was no difference in renal function between those who had continued or stopped their ACEi/ARB (mean eGFR in continued group 13.3 ± 0.6 mL/min/1.73 $m^{2}$ vs discontinued group 12.6 ± 0.7 mL/min/1.73 $m^{2}$; 95% CI –2.5–1.0*; p = *0.42) [83]. Furthermore, there was a trend, albeit not statistically significant, to fewer cardiovascular events in the continued RAASi arm (n = 88), than those who discontinued (n = 108).

Thus, increasing evidence suggests that an initial increase in creatinine of up to 30% should be viewed similarly to a reduction in pulse rate upon commencing beta-blockers; a direct consequence of the medication, with no long-term deleterious effects [9, 84]. However, a larger increase in serum creatinine or a deterioration in the clinical status of the patient should prompt a thorough assessment by a clinician to rule out alternative explanations such as renal artery stenosis and hypovolemia.

#### 4.1.5 ACEi/ARB and Hyperkalaemia 

ACEi’s/ARB’s also increase the likelihood of hyperkalaemia (serum potassium >5.5 mmol/L) [85]. This is a particular concern because as the eGFR declines, the risk of hyperkalaemia increases and can be fatal [86]. There have been previous studies outlining the potential of potassium binding agents such as sodium zirconium cyclosilicate or Patiromer to reduce potassium levels in patients with CKD or HF [87]. The UK National Institute for Health and Care Excellence (NICE) guidelines recommend the use of sodium zirconium cyclosilicate in patients with CKD stages G3b-5 or HF whose hyperkalaemia (serum potassium >6 mmol/L) prohibit them from using optimal RAASi doses [88]. There is an ongoing RCT to investigate its use within the unique cohort of patients with both CKD and HF [86]. Physicians should refer to the 2021 International Society of Nephrology (2021) toolkit on the optimisation of RAASi therapy for guidance regarding rechallenging medication following acute kidney injury or hyperkalaemia [89].

#### 4.1.6 ACEi/ARB Summary 

In summary, there is consistent and strong evidence for ACEi/ARB in HFrEF and CKD stages G1-3. Further evidence is needed in CKD stages G4/5 CKD and in HFmrEF. There is currently no role for ACEi/ARB in HFpEF. Serum creatinine, potassium and blood pressure should be closely monitored when RAASi is commenced and up titrated, especially in those with CKD. An increase of serum creatinine of up to 30% is both acceptable and expected and should not, alone, be a reason for RAASi withdrawal. Potassium binders may be used where hyperkalaemia consistently prohibits up titration of RAASi.

### 4.2 ARNI 

#### 4.2.1 ARNI in HFrEF 

Neprilysin is an endopeptidase which breaks down naturally occurring vasoactive peptides. Using the drug, sacubitril, to inhibit neprilysin leads to greater circulating levels of vasoactive peptides including natriuretic peptides and bradykinin, leading to natriuresis and vasodilatation and counteracting the negative consequences of RAAS activation [30]. Sacubitril has been used in combination with ARB’s such as valsartan, to form a new class of medical-therapy for HF called ARNI’s, such as Sacubitril/valsartan. Although the first trial demonstrating the efficacy of Sacubitril/valsartan was published in 2014 (PARADIGM-HF) and it was approved by the Food and drug administration (FDA) in 2015, its implementation has been slow, with a US study of 3518 patients published in 2018 showing that only 13% of eligible patients were receiving ARNI [10, 90].

The PARADIGM-HF trial of 4187 ambulatory patients showed that Sacubitril/valsartan led to reduced HF hospitalisation or death from cardiovascular cause, compared to enalapril (HR 0.80; 95% CI 0.73–0.87; *p* < 0.001) [30]. Patients treated with Sacubitril/valsartan were also less symptomatic at 8 months (*p* = 0.001) and experienced less death from any cause (HR 0.84; 95% CI 0.76–0.93; *p* < 0.001) [30]. Additionally, Sacubitril/valsartan was better tolerated than enalapril, with fewer patients discontinuing their medication due to an adverse event (10.7% vs 12.3%, *p* = 0.03), including renal impairment (0.7% vs 1.4%, *p* = 0.002). The PIONEER-HF (Comparison of Sacubitril/valsartan versus Enalapril on Effect on N-terminal pro-B-type natriuretic peptide (NT-proBNP) in Patients Stabilized from an Acute HF Episode) trial demonstrated that the addition of Sacubitril/valsartan in patients hospitalised with acute HF led to significantly greater NT-proBNP reductions compared with enalapril therapy (ratio of change 0.71; 95% CI 0.63–0.81; *p <* 0.001) [28].

In both trials, patients with CKD stages G4-5 were excluded (Table 6, Ref. [28, 29, 30]). However, a subgroup analysis in PIONEER-HF suggested that the benefit of Sacubitril/valsartan was consistent regardless of mild (stage G2-3) baseline renal impairment [28]. In 2016, ESC guidelines recommended either an ARNI or ACEi should be used alongside MRA or β-blockers to treat patients with HFrEF. They recommended ARNI as a replacement for ACEi in patients with HFrEF who remain symptomatic despite management with ACEi, beta-blocker and MRA, to reduce further the risk of death and HF hospitalization [1].

**Table 6.** **Summary of pivotal RCT’s for use of ARNIs for management of HF**.

Trial name, year (Ref)	N	Main outcome	Intervention *(target dose) *vs comparator *(target dose*)	LVEF inclusion criteria	Renal exclusion criteria	NYHA class of participants	Overall results (Primary outcome) (95% CI; *p* value)
PARADIGM-HF, 2014 [30]	8442	Composite of death from CVS causes and hospitalisation for HF	Sacubitril/valsartan *(97 mg/103 mg BD)* vs enalapril *(10 mg BD)*	Initially ≤40%, changed to ≤35%	eGFR <30 mL/min/1.73 $m^{2}$	I – 4.6%	HR 0.80 (95% CI 0.73 to 0.87; *p * < 0.001)
						II – 70.5%	
						III – 24%	
						IV – 0.7%	
						Missing – 0.2%	
PARAGON-HF, 2019 [29]	4796	Composite of death from CVS causes and hospitalisation for HF	Sacubitril/valsartan *(97 mg/103 mg BD)* vs valsartan *(160 mg BD)*	≥45%	eGFR <30 mL/min/1.73 $m^{2}$	I – 2.9%	Rate ratio 0.87 (95% CI 0.75–1.01; *p* = 0.06)
						II – 77.3%	
						III – 19.4%	
						IV – 0.4%	
						Missing – 0.04%	
PIONEER, 2019 [28]	881	Time-averaged proportional change in NT-proBNP	Sacubitril/valsartan *(97 mg/103 mg BD)* vs enalapril *(10 mg BD)*	≤40%	eGFR <30 mL/min/1.73 $m^{2}$	I – 1.0%	Ratio of change 0.71 (95% CI 0.63 to 0.81; *p * < 0.001)
						II – 25.2%	
						III – 62.7%	
						IV – 8.5%	
						Missing – 2.6%	

Abbreviations used in Table 6: ARNI, angiontensin receptor neprilysin inhibitor; BD, twice a day; CI, confidence interval; CVS, cardiovascular; eGFRm, estimated glomerular filtration rate; HF, heart failure; HR, hazard Ratio; LVEF, left ventricular ejection fraction; m, metre; mg, milligram; min, minute; mL, millilitre; NT-proBNP, N-terminal pro B-type natriuretic peptide; NYHA, New York Heart Association Classification; PARADIGM-HF, prospective comparison of ARNI with ACEI to determine impact on global mortality and morbidity in heart failure; PARAGON-HF, prospective comparison of ARNI with ARB global outcomes in HF with preserved ejection fraction; PIONEER, comparison of sacubitril/valsartan versus enalapril on effect on NT-proBNP in Patients stabilized from an acute HF episode; RCT, randomised controlled trial; eGFR, estimated glomerular filtration rate.

#### 4.2.2 ARNI in HFmrEF 

No trial has yet specifically investigated ARNI use in HFmrEF. However, analysis of other studies which include patients with LVEF 41–49% provide some indication that ARNI may be beneficial, especially in reducing HF hospitalisations, for patients with HFmrEF [29, 91]. The ESC 2021 HF guidelines recommend that ARNI may be considered for these patients based on this Class IIb evidence [1].

#### 4.2.3 ARNI in HFpEF 

The PARAGON-HF trial evaluated Sacubitril/valsartan vs valsartan in 4822 patients with HFpEF, and found reduced rates of the composite primary outcome of total hospitalisations for HF and death from cardiovascular causes (rate ratio 0.87), albeit this narrowly missed statistical significance (95% CI 0.75–1.01; *p* = 0.06) [29]. However, sub-group analysis of patients with eGFR <60 mL/min/1.73 $m^{2}$, did reach statistical significance for this primary outcome in favour of ARNI [29]. Although patients with severe renal impairment were excluded and further evidence is required for this cohort, this provides evidence that patients with HFpEF and mild renal impairment may benefit from ARNI. Furthermore, post-hoc analyses suggested that certain subgroups within the HFpEF population were likely benefit from ARNI e.g., patients with raised troponin, recent hospitalisation due to HF, or in those previously established on MRA; likely reflective of the heterogeneity of pathology encapsulated within the subgroup of HFpEF [92, 93, 94].

#### 4.2.4 Side-Effects of ARNI 

Similarly, to ACEi and ARB, there is often a reversible increase in creatinine when ARNIs are commenced or titrated. However, RCT’s and observational studies have all found that ARNIs are superior to ACE/ARB in protecting renal function [10, 30, 95, 96]. A meta-analysis including 16,456 patients from ten RCT’s, showed a 30% reduced risk of renal impairment with ARNI compared to ACE/ARB (Pooled OR 0.70; 95% CI 0.57–0.85; *p* < 0.001); which was even greater in patients with HFpEF [97]. The survival benefits with these drugs outweigh any transient decline in renal function on commencing them, and as with ACEi/ARB, these medications should not be unnecessarily paused or withheld for a mild reduction in renal function alone [10].

PARAGON-HF and PARADIGM-HF also demonstrated that hyperkalaemia was significantly less common in patients taking ARNI than ACEi/ARB [29, 30].

A systematic review and meta-analysis of six studies involving 6217 patients suggests that patients with CKD are more likely to experience hypotension when taking ARNI than those without CKD, however, this effect was dose-dependent and predictable [24].

#### 4.2.5 ARNI Summary 

In summary, ARNI have been shown to be effective for HFrEF, HFmrEF and less likely to cause renal impairment or hyperkalaemia, and better tolerated compared with ACEi or ARB. Blood pressure and renal function should be monitored when commencing these medications. Although not HF specific, a recent RCT used ARNI in 207 patients with an average eGFR of 34.0 mL/min/1.73 $m^{2}$, (lowest eGFR 20 mL/min/1.73 $m^{2}$) over a 12-month period with no major safety concerns. However, as there has been little research in patients with severe CKD, more trials are required to confirm the safety and efficacy in this cohort [98].

## 5. MRA

Mineralocorticoid receptors (MR) are another key RAAS player. Classically MR are expressed in the “aldosterone-sensitive” collecting duct epithelium, facilitating renal sodium resorption and excretion of potassium. Non-classical expression of MR on podocytes, cardiac myocytes, fibroblasts, endothelium and vascular smooth cells can lead to pathological changes in the heart including cardiac remodelling, fibrosis and may contribute to arrythmias. In the kidneys activation of these receptors can lead to glomerular and tubular sclerosis and fibrosis [99, 100].

Since spironolactone was introduced as the first MRA in 1959, the more selective eplerenone and recently non-steroidal MRAs such as finerenone have become available and accepted into clinical practice, changing the scope of care for diabetic kidney disease. Whilst MRAs form one of the pillars of the recommended quadruple therapy for management of chronic HFrEF, concerns regarding worsening renal function and hyperkalemia in context of HF in CKD, usually complicated by frailty and polypharmacy have limited their use in this population. As such many, trials on MRAs in HF have traditionally excluded patients with advanced CKD (eGFR <30 mL/min/1.73 $m^{2}$) (Table 7, Ref. [31, 101, 102, 103]), and much of the evidence supporting their use in this context comes from sub-group and post-hoc analysis.

**Table 7.** **Summary of pivotal RCT’s for use of MRA’s for management HF**.

Trial name, year (Ref)	N	Main outcome	Intervention *(target dose) *vs comparator *(target dose)*	LVEF inclusion criteria	Renal exclusion criteria	NYHA class of participants	Overall results (Primary outcome) (95% CI; *p* value)
RALES, 1999 [101]	1663	All-cause mortality	Spironolactone *(25 mg OD)* vs placebo	≤35%	Creatinine >221 µmol/L (2.5 mg/dL)	II – 0.4%	35% vs 46%; RR 0.70 (95% CI 0.60–0.82; *p * < 0.001)
						III – 70.5%	
						IV – 29%	
EMPHASIS-HF, 2011 [31]	2737	Composite of cardiovascular death or HF hospitalisation	Eplerenone *(50 mg OD)* vs placebo	≤35%	eGFR <30 mL/min/1.73 $m^{2}$	II – 100%	18.3% vs 25.9%; HR 0.63 (95% CI 0.54–0.74; *p * < 0.001)
TOPCAT, 2014 [102]	1722	Composite of cardiovascular death, aborted cardiac arrest or HF hospitalisation	Spironolactone *(45 mg OD) *vs placebo	≥45%	eGFR <30 mL/min/1.73 $m^{2}$ OR Creatinine >221 µmol/L (2.5 mg/dL)	I – 3.2%	18.6% vs 20.4%; HR 0.89 (95% CI 0.77–1.04; *p* = 0.14)
						II – 63.7%	
						III – 32.5%	
						IV – 0.4%	
						Missing – 0.2%	
ATHENA-HF, 2017 [103]	360	Change in NT-proBNP levels at 96 hours	Spironolactone *(100 mg OD)* vs placebo/spironolactone (*25 mg OD)*	None. Median baseline 34%. 26% had LVEF >45%	eGFR <30 mL/min/1.73 $m^{2}$	III/IV – 83.9%	–0.49 (–0.98 to –0.14) vs –0.55 (–0.92 to –0.18), *p* = 0.57

Abbreviations used in Table 7: CI, confidence interval; ATHENA, aldosterone targeted neurohormonal combined with natriuresis therapy in heart failure; dL, decilitre; eGFR, estimated glomerular filtration rate; EMPHASIS, eplerenone in mild patients hospitalization and survival study in heart failure; HF, heart failure; HR, hazard Ratio; L, litre; LVEF, left ventricular ejection fraction; m, metre; mg, milligram; min, minute; mL, millilitre; MRA, mineralocorticoid receptor antagonists; NT-proBNP, N-terminal pro B-type natriuretic peptide; NYHA, New York Heart Association Classification; OD, once a day; RALES, randomized aldactone evaluation study; RCT, randomised controlled trial; RR, relative risk; TOPCAT, treatment of preserved cardiac function heart failure with an aldosterone antagonist.

### 5.1 MRA in HFrEF 

The Randomized aldactone evaluation study (RALES) study was the first trial of an MRA (spironolactone) versus placebo in patients with HFrEF on standard therapy (including ACEi, digoxin and diuretics, with only a small proportion of both trial and placebo arm on beta blockers) [101]. The trial, including 1663 patients, was stopped early after a mean follow up of 24 months due to the significant mortality benefit observed [101]. There was a 30% reduction in the risk of death observed in the spironolactone group compared to placebo (95% CI 0.60–0.82, *p* < 0.001), in addition to a 35% decrease in the hospitalisations due to worsening HF (95% CI 0.54–0.77, *p* < 0.001).

In the sub-group analysis of patients with eGFR <60 mL/min/1.73 $m^{2}$, spironolactone had a similar risk reduction for all-cause death and combined endpoint of hospital stays due to worsening HF or death compared to patients with eGFR >60 mL/min/1.73 $m^{2}$. The risk of worsening renal function (>30% decrease in eGFR) and hyperkalemia was greater in patients with underlying poor renal function, but the mortality benefit of spironolactone therapy was maintained [104].

Eplerenone was observed to have significant mortality benefit when the EMPHASIS-HF (eplerenone in mild patients hospitalisation and survival study in HF) study was stopped at 21 months of mean follow up, showing a 37% decrease in combined primary end point of hospitalisations due to HF of death due to cardiovascular causes compared to placebo [31]. A sub group analysis in patients with eGFR 30–60 mL/min/1.73 $m^{2}$, age ≥75 years, diabetes and systolic blood pressure <123 mmHg (deemed to be at high risk of developing worsening renal function and hyperkalemia) found a reduction in primary composite end-point across all sub-groups with eplerenone [105]. However there was a greater incidence of hyperkalemia (serum potassium >5.5 mmol/L), and hospital admissions due to hyperkalemia and discontinuation of therapy due to hyperkalemia; there was no increased incidence of severe hyperkalemia (>6.0 mmol/L) [105].

The ARTS (MinerAlocorticoid Receptor Antagonist Tolerability Study), was a phase II RCT conducted in two parts to evaluate the tolerability and safety of finerenone [106, 107]. In Part A the use of finerenone was compared with placebo in patients with HFrEF and mild CKD (eGFR 60–90 mL/min/1.73 $m^{2}$), whereas in part B finerenone use was compared to placebo and spironolactone group in patients with HFrEF and moderate CKD (eGFR 30–60 mL/min/1.73 $m^{2}$). Finerenone was found to cause a smaller increase in serum potassium concentration compared to spironolactone, and consequently less incidence of hyperkalemia and worsening renal function. It caused a similar reduction in BNP, NT-proBNP and albuminuria compared to spironolactone, with a safer side-effect profile [106, 107].

Finerenone was compared to eplerenone to evaluate the efficacy and safety in patients with HFrEF with CKD (eGFR 30–60 mL/min/1.73 $m^{2}$ in patients without diabetes) and/or Type 2 diabetes (eGFR >30 mL/min/1.73 $m^{2}$). Compared with eplerenone, the composite endpoint (all-cause mortality, hospitalisation due to cardiovascular causes or worsening HF) was lower in all finerenone groups with dose >2.5–5 mg at 90 days. There was lower incidence of hyperkalemia and worsening renal failure in the finerenone group, compared to the eplerenone group [108, 109].

An observational single-centre Swedish study by Holmdahl *et al*. [110], retrospectively analysed the outcomes of 416 patients with HFrEF and moderate CKD (eGFR <60 mL/min/1.73 $m^{2}$); 131 of whom were prescribed MRA (age 77 ± 9 years), and 285 of whom were not (age 82 ± 9 years). It was observed that the use of MRA in elderly patients with HFrEF and moderately impaired renal function was not associated with worsening renal function, and did not impact all-cause mortality [110].

### 5.2 MRA in HFmrEF and HFpEF 

The use of MRA (Spironolactone vs placebo) in HF patients with LVEF ≥45% was investigated in the Treatment of Preserved Cardiac Function Heart Failure With an Aldosterone Antagonist (TOPCAT) trial (Spironolactone for Heart Failure with Preserved Ejection Fraction), which found no difference between the two arms in terms of the primary outcome (time to death due to cardiovascular causes, hospitalisation due to HF and/or aborted cardiac arrest) [102]. Curiously, spironolactone was observed to be superior to placebo in terms of this primary outcome in patients recruited from Americas [111]. A post-hoc analysis of this sample stratified further based on renal function (eGFR ≥60, 45–59 and <45 mL/min/1.73 $m^{2}$) observed that the effect of spironolactone was similar across all groups, however, worsening renal function was associated with worsening renal function and hyperkalemia. Authors concluded that for every 100 patients with HFpEF treated with spironolactone, nine primary outcome events would be prevented however it would lead to 27 events of terminating medication use [112]. As this trial did not reach its primary endpoint, it should be viewed as hypothesis generating only, and at present, guidelines do not recommend the use of MRA in patients with HFpEF. MRA may be considered in HFmrEF with close monitoring [1].

### 5.3 MRA Summary 

A systemic review by Khan *et al*. [113] in 2020 including seven studies (three in HFrEF, one in HFpEF, two with acute decompensated HF and one with mixed HF population) concluded that MRA use in patients with CKD (eGFR 30–60 mL/min/1.73 $m^{2}$) was associated with reduced risk of primary end point (hospitalisation due to HF, all-cause mortality and adverse cardiovascular outcomes). However, there was higher risk of developing hyperkalemia and consequent discontinuation of medication.

Furthermore, there have been recent promising suggestions of non-steroidal MRA’s role in the primary prevention of HF in patients with CKD and type 2 diabetes. A post-hoc analysis of the Finerenone in Reducing Cardiovascular Mortality and Morbidity in Diabetic Kidney Disease (FIGARO-DKD) trial suggested that finerenone significantly reduced the risk of incident HF by 32% in patients with diabetic kidney disease [114]. The Combined FIDELIO-DKD and FIGARO-DKD Trial programme (FIDELITY) analysis similarly demonstrated that finerenone significantly reduced first hospitalisation for HF in patients with CKD and type 2 diabetes [115].

In conclusion, while MRA remains an important pillar of HFrEF treatment, caution should be exercised in the complex patient group with both CKD and HF, usually complicated with frailty, multimorbidity and polypharmacy, and close biochemical monitoring is important during treatment. Further evidence is required for HFmrEF and HFpEF, but MRA may be considered in patients with HFmrEF with close monitoring.

## 6. Beta Blockers 

Beta-blockers form one of the 4 main pillars of treating HF; they work by reducing stress on cardiac muscle from sympathetic de-activation, thereby improving LVEF [9]. Numerous pivotal RCT’s with large patient numbers have demonstrated the efficacy of beta-blockers in reducing all-cause mortality and hospitalisation compared to placebo in patients with HFrEF and HFmrEF (Table 8, Ref. [116, 117, 118, 119, 120, 121, 122, 123, 124, 125]). Post-hoc sub-group analyses of these trials based on renal function are concordant with the efficacy of beta-blockers in improving outcomes of patients with kidney disease, regardless of the severity of renal impairment. Beta-blockers are effective across the drug-class, with no one clear superior agent, according to one meta-analysis in patients with HFrEF [126].

**Table 8.** **Summary of pivotal RCT’s for use of beta-blockers for management of HF**.

Trial name, year (Ref)	N	Main outcome	Intervention *(target dose) *vs comparator *(target dose)*	LVEF inclusion criteria	Renal exclusion criteria	NYHA class of participants	Overall results (Primary outcome) (95% CI; *p* value)
CIBIS II [116, 117]	2647	All-cause mortality	Bisoprolol *(1.25 mg OD) *vs placebo	<35%	≥300 µmol/L	III – 83.2%	11.8% vs 17.3%; HR 0.66 (95% CI 0.54–0.81; *p * < 0.0001)
						IV – 17.1%	
COPERNICUS, 2001 [118]	2289	All-cause mortality	Carvedilol *(25 mg BD)* vs placebo	<25%	>247.5 µmol/L	II–IV (proportions not stated)	12.8% vs 19.7%; RR 0.65 (95% CI 0.52–0.81; *p* = 0.00013)
MERIT HF, 1999 [119, 120]	3991	All-cause mortality	Metoprolol controlled release/extended release (CR/XL) *(12.5–25 mg OD)* vs placebo	<40%	N/A	II – 41.0%	7.2% vs 11.0% per patient–year of follow–up; RR 0.66 (95% CI 0.53–0.81; *p* = 0.00009)
						III – 55.4%	
						IV – 3.6%	
SENIORS, 2009 [121]	2128	Composite outcome of all-cause mortality or cardiovascular hospitalisation	Nebivolol *(10 mg OD) *vs placebo	<35%	≥250 µmol/L	I – 2.9%	31.1% vs 35.3%; HR 0.86 (95% CI 0.74–0.99; *p* = 0.039)
						II – 56.4%	
						III – 38.7%	
						IV – 2.0%	
COMET, 2003 [122]	3029	(1) All-cause mortality	Carvedilol *(25 mg BD)* vs metoprolol *(50 mg BD)*	<35%	N/A	II – 48.4%	(1) 34% vs 40%; HR 0.83 (95% CI 0.74–0.93; *p* = 0.0017)
		(2) Composite outcome of all-cause mortality or all-cause admission				III – 47.8%	(2) 74% vs 76%; HR 0·94 (95% CI 0.86–1.02; *p* = 0.122)
						IV – 3.8%	
Carvedilol US, 1996 [123]	1094	All-cause mortality	Carvedilol *(50 mg BD)* vs placebo	≤35%	N/A	II – 53.2%	3.2% vs 7.8%; Risk Reduction 65% (95% CI 39–80%; *p * < 0.001)
						III – 43.9%	
						IV – 2.9%	
CAPRICORN, 2001 [124]	1959	(1) All-cause mortality	Carvedilol *(25 mg BD)* vs placebo	≤40%	N/A	N/A	(1) 12% vs 15%; HR 0.77 (95% CI 0.60–0.98; *p* = 0.031)
		(2) Composite outcome of all-cause mortality or cardiovascular hospitalisation					(2) 35% vs 37%; HR 0.92 (95% CI 0.80–1.07; *p* = 0.296)
BEST, 2001 [125]	2708	All-cause mortality	Bucindolol *(100 mg BD) *vs placebo	≤35%	≥265 µmol/L	III – 91.7%	33% vs 30%; HR 0.90 (95% CI 0.78–1.02; *p* = 0.13)
						IV – 8.3%	

Abbreviations used in Table 8: BD, twice a day; CI, confidence interval; HF, heart failure; HR, hazard ratio; LVEF, left ventricular ejection fraction; mg, milligram; NYHA, New York Heart Association Classification; OD, once a day; RCT, randomised controlled trial; RR, relative risk; µmol,micromol; CIBIS, cardiac insufficiency bisoprolol study; COPERNICUS, carvedilol prospective randomized cumulative survival; MERIT, metoprolol CR/XL randomised intervention trial in congestive heart failure; SENIORS, study of effects of nebivolol intervention on outcomes and rehospitalization in seniors with heart failure; COMET, carvedilol or metoprolol european trial; CAPRICORN, effect of carvedilol on outcome after myocardial infarction in patients with left-ventricular dysfunction; BEST, beta-blocker evaluation of survival trial; CR, controlled release; XL, extended.

Meta-analyses combining results of post-hoc renal impairment stages from pivotal trials demonstrated that beta-blockers reduced risk of death across all stages of CKD [127, 128, 129]. In a large meta-analysis of 16,740 patients, eGFR was found to independently affect mortality (12% higher risk of death for every 10 mL/min/1.73 $m^{2}$ lower eGFR), and with higher mortality at follow-up as renal function worsened; but beta-blockers reduced mortality compared to placebo [128]. Another meta-analysis of 4217 patients reported carvedilol only transiently increased creatinine in the serum without requiring haemofiltration, and was notably insignificant in CKD stage G3b [127].

However, clinical trials have noted greater discontinuation of beta-blockers in this cohort of CKD-HF patients, mainly due to intolerance from bradycardia. Renal impairment in patients with HF pre-disposes to up-regulated action of various biomechanisms; notions suggested include up-regulation of the renin-aldosterone system which results in worsening inflammation, stress, and vasoconstriction [130, 131, 132]. Practically, patients with HF should be initiated on beta-blocker therapy at the highest dose tolerated and should be monitored for heart rate [1, 133]. Studies assessing efficacy of beta-blocker use in patients with CKD and HFpEF are limited [134].

## 7. SGTL2i 

As of the 2023 ESC HF Guideline update, SGTL2i’s are now recommended for patients with HF with any ejection fraction [37]. SGLT2i are cardioprotective and renoprotective in several ways; they inhibit the glomerular hyperfiltration occurring in type 2 diabetes mellitus (commonest risk factor for CKD), due to their enhanced tubule-glomerular feedback. Additionally, they reduce the energy consumption of the sodium-glucose transporter by inhibiting it, therefore protecting the kidney from hypoxia, which is a common pathway for the progression of CKD [135]. Their cardioprotective mechanisms include reduced afterload and improved cardiac blood flow [136].

### 7.1 SGLT2i in HFrEF 

The pivotal trials to demonstrate benefits of SGLT2i’s in HFrEF were: DAPA-HF (The Dapagliflozin and Prevention of Adverse Outcomes in HF) [25], EMPEROR-Reduced (Empagliflozin Outcome Trial in Patients with Chronic HF and Reduced Ejection Fraction) [26], and SOLOIST-WHF (The effect of Sotagliflozin on Cardiovascular Events in Patients with Type 2 Diabetes Post Worsening HF) [27]. The DAPA-HF study (2019) showed that dapagliflozin was associated with a reduced risk of progressive HF or cardiovascular death relative to placebo in 4744 patients (HR 0.74; 95% CI 0.65–0.85; *p* < 0.001) [25].

The following year, Empagliflozin Outcome Trial in Patients With Chronic Heart Failure and Reduced Ejection Fraction (EMPEROR-Reduced) replicated these findings in 3730 patients, this time using empagliflozin vs placebo (HR 0.75; 95% CI 0.65–0.86; *p* < 0.001) [26].

These studies all excluded patients with severe renal impairment (eGFR of 20 mL/min/1.73 $m^{2}$ in EMPEROR-Reduced and 30 mL/min/1.73 $m^{2}$ in DAPA-HF and SOLOIST-WHF), however, up to CKD stage G3b there is good evidence for their use with no evidence of harm. Furthermore, EMPEROR-Reduced included 204 patients with CKD stage G4 at baseline, and the same cardiovascular and renal benefits were observed across the following eGFR subgroups: >90, 60 to <90, 45 to <60, 30 to <45 and <30 mL/min/1.73 $m^{2}$, with no evidence of any harm [26].

### 7.2 SGLT2i in HFmrEF and HFpEF 

In the 2023 ESC HF Guideline update, the recommendations for SGLT2i’s were extended to HFmrEF and HFpEF, based on Class I evidence of their ability to reduce risk of cardiovascular death or HF hospitalisation within these population. This was largely due to two clinical trials; EMPEROR-Preserved published in 2021 [18] and Dapagliflozin Evaluation to Improve the Lives of Patients with Preserved Ejection Fraction Heart Failure (DELIVER) in 2022 [19]. EMPEROR-Preserved was a multi-centre phase III RCT which randomised 5988 patients with HF and LVEF >40% (median LVEF 54%) to receive either empagliflozin (target dose 10 mg OD) or placebo. At median 26.2 months, patients treated with empagliflozin had 21% lower event rates (cardiovascular death or hospitalisation with HF) than patients on placebo (HR 0.79; 95% CI 0.69–0.90; *p* < 0.001). This reduced event rate was consistent across those with or without diabetes [18]. The DELIVER trial then demonstrated a similar 18% risk reduction in primary outcome in patients with HF and LVEF >40% using dapagliflozin vs placebo, (HR 0.82; 95% CI 0.73–0.92; *p* < 0.001) [19]. In both trials, the risk reduction was determined primarily by a significant risk reduction in hospitalisations for HF. When examined independently, risk of cardiovascular death was not significantly reduced. A meta-analysis including these studies showed that the benefits of SGLTi were seen across the spectrum of LVEF >40% suggesting benefit of its use in both HFmrEF and HFpEF [137].

Renal exclusion criterion for EMPEROR-Preserved and DELIVER were eGFR <20 and 25 mL/min/1.73 $m^{2}$, (as per the Chronic Kidney Disease Epidemiology Collaboration [CKD-EPI] equation), respectively. In both trials, approximately half the participants had an eGFR of <60 mL/min/1.73 $m^{2}$ and the benefit of SGLT2i was maintained across both patients with and without CKD.

Furthermore, in EMPEROR-Preserved, nearly 10% had an eGFR of <30 mL/min/1.73 $m^{2}$ and empagliflozin reduced the decline in kidney function across the spectrum of baseline eGFR [18].

### 7.3 Side-Effects of SGLT2i 

Similarly to ACEi/ARB/ARNI, when commencing or titrating SGLT2i’s, there can be an initial apparent worsening in kidney function (e.g., in the DAPA-CKD trial, patients in the dapagliflozin group had an eGFR decline at 2 weeks of –2.10 (0.37) vs 0.68 (0.35) mL/min/1.73 $m^{2}$ in the placebo group, *p* = 0.005). However, DAPA-CKD demonstrated that beyond this initial drop, patients treated with dapagliflozin had a less steep eGFR decline per year than those on placebo (1.23 vs 1.73 mL/min/1.73 $m^{2}$ per year, *p* = 0.005). This was seen even in the cohort of patients with CKD stage G4 [138]. This was confirmed in the Empagliflozin, Cardiovascular Outcomes, and Mortality in Type 2 Diabetes trial (EMPA-REG) [139], The Study of Heart and Kidney Protection With Empagliflozin (EMPA-KIDNEY) [140] and EMPEROR-Preserved [18] studies, with EMPA-REG confirming that this initial ‘eGFR dip’ did not impact patients’ long term renal or cardiovascular outcomes.

Other known side-effects of SGLTi, which can preclude their use, include recurrent urinary tract infections and diabetic ketoacidosis (DKA). The Sotagliflozin in Patients with Chronic Kidney Disease and Type 2 Diabetes (SCORED) trial (2021) was a multi-centre RCT which compared sotagliflozin to placebo in 10584 patients with CKD (eGFR 25–60 mL/min/1.73 $m^{2}$) and type 2 diabetes mellitus [27]. It found that patients randomised to SGLTi, when compared to placebo, had significantly higher rates of diarrhoea (8.5% vs 6.0%, *p* < 0.001) volume depletion (5.3% vs 4.0%, *p* = 0.003), genital mycotic infections (2.4% vs 0.9%, *p* < 0.001) and diabetic ketoacidosis (0.6% vs 0.3%, *p* = 0.02). The trial found the SGLT2i led to a lower risk of composite of heart failure hospitalisation, cardiovascular death and urgent hospital visit for HF, when compared to placebo [27].

This review focuses primarily on chronic HF; however, of note, a recent meta-analysis [141] of three randomised controlled trials in acute HF populations (SOLOIST [27], The SGLT2 inhibitor empagliflozin in patients hospitalized for acute heart failure (EMPULSE) [142] and The effects of empagliflozin on clinical outcomes in patients with acute decompensated heart failure (EMPA-RESPONSE-AHF) [143]) found that in patients hospitalised with acute HF, SGLT2i reduced all-cause and cardiovascular mortality compared to placebo. Furthermore, there were low rates of adverse events. In SOLOIST, there were 2 cases of diabetic ketoacidosis in the SGLT2i group (0.3%), compared to 4 in the placebo group (0.7%) [27]. In EMPULSE ketoacidosis occurred in none of the 530 participants [142]. These trials confirm that SGLT2i are both effective and safe in acute HF.

### 7.4 SGLT2i Summary 

The efficacy of SGLT2i is consistent amongst various patient groups; regardless of diabetic status, LVEF, and variation in severity of CKD (demonstrated up to eGFR <20 mL/min/1.73 $m^{2}$). Consequently, it is now recommended in all classes of HF, and has become the first evidence-based medical therapy for HFpEF [144]. More research is needed on the safety and efficacy of these medications in stage G5 CKD and in patients on haemodialysis. Furthermore, although there are some serious side-effects associated with their use, these are rare and there are steps which can be taken to mitigate the risk (Table 9). Sick day rules and other things to remember for prescribing SGLT2-I’s can be found in **Appendix**.

**Table 9.** **Summarises the main known side-effects of SGLT2i’s and ways to mitigate each of these risks**.

Side effect	Management
Hypoglycemia is common when used with insulin	At initiation, reduce the dose of sulfonylurea or insulin if eGFR >45 mL/min/1.73 $m^{2}$ and glycated hemoglobin (HbA1c) <58 mmol/mol
Urinary tract infections (UTI) may happen	Use with caution in patients with poor urinary flow and bladder outlet obstruction
	Serious UTIs such as urosepsis and pyelonephritis may occur with SGLT2i use and this is where it needs to be stopped prior to further evaluation. Evaluate and treat as needed, and dependent on severity.
Vulvovaginal infections are usually mild and resolve with appropriate treatment	Supportive treatment and address modifiable risk factors including optimizing diabetes care and personal hygiene.
Dyslipidemia - small increase in LDL-C and HDL levels can occur with SGLT2i use	Monitor lipid profile and treat as necessary
Back pain is benign	Rule out malignancy and fractures, and manage as needed
Diabetic ketoacidosis (DKA) The risk for DKA is highest for canagliflozin, followed by empagliflozin and dapagliflozin	Consider risk factors that may predispose patient to DKA prior to initiation and if DKA occurs, discontinue the SGLT2i, and evaluate and treat promptly
Necrotising fasciitis/Fournier’s gangrene is a rare but serious side effect of SGLT2i	Urgent surgical assessment and treatment and discontinue SGLT2i
Peripheral vascular disease and amputation risk	Avoid SGLT2i initiation in the presence of active foot infection, ulceration or ischemia. Withhold SGLT2i in those who develop foot disease during treatment and restart treatment following resolution
Angioedema and other hypersensitivity reactions such as erythema, rash, pruritus, and angioedema are rare	Discontinue the SGLT2i and monitor until signs and symptoms resolve. Hypersensitivity reactions such as anaphylaxis or angioedema would be a contraindication to any further future use
Hypovolemia and acute kidney injury is more likely to occur especially in those receiving diuretics and those with CKD prior to SGLT2i initiation	Early clinical review and reduction of diuretic doe is recommended. SGLT2i may need to be withheld if hypovolemia is associated with acute illness. Evaluate if SGLT2i should be stopped on a case-to-case basis in AKI [see sick day rules]

Abbreviations used in Table 9: AKI, acute kidney injury; CKD, chronic kidney disease; DKA, diabetic ketoacidosis; eGFR, estimated glomerular filtration rate; HbA1c, glycated hemoglobin; HDL, high-density lipoprotein; LDL-C, low density lipoprotein cholesterol; SGLT2i, sodium-glucose cotransporter-2 inhibitors.

## 8. Others 

### 8.1 Digoxin 

Digoxin is one of the oldest compounds used in HF. It is a cardiac glycoside that is derived from the foxglove plant and originally described by William Withering in 1785 [145]. Digoxin exerts a positive inotropic and negative chronotropic effect on the heart, by binding to the ${\mathrm{Na}}^{+}$-$K^{+}$ ATPase pump [146]. Digoxin has a narrow therapeutic interval and requires tight monitoring, especially in patients with renal impairment. In a pharmacokinetic study for digoxin in patients with HF and CKD, Lin *et al*. [147] demonstrated that a reduced dosage regimen adjusted for a patient’s eGFR, dose of metoprolol, and body weight, would achieve a higher probability of target attainment.

#### 8.1.1 Digoxin in HFrEF 

In the Digitalis Investigation Group (DIG) multi-centre RCT, digoxin was compared to placebo in patients with HF with LVEF <45%, in sinus rhythm and with serum creatinine levels <3.0 mg/dL (265 µmol/L). This corresponds to a renal function cut off of eGFR 20 mL/min/1.73 $m^{2}$ [148]. In a mean follow up of 37 months (range 28–58), digoxin had no effect on all-cause mortality (RR 0.99; 95% CI 0.76–1.28; *p* = 0.925), but was shown to reduce HF hospitalisations (RR 0.72; 95% CI 0.66 to 0.79; *p* < 0.001). In a secondary analysis of the DIG trial, Shlipak *et al*. [149] showed that the effect of digoxin was comparable across eGFR subgroups.

Since the DIG trial was published, various observational studies have shown increased mortality and hospitalisation rate with patients on digoxin compared to those not on digoxin in patients with HFrEF [150, 151]. This is similarly shown in patients with advanced kidney disease [152, 153]. The hypothesis regarding the difference in effect is that a prescription bias exists; digoxin is more often prescribed to patients with advanced HF in clinical practice, compared to in a RCT. A secondary analysis of the DIG trial compared the baseline characteristics of those who were treated with digoxin prior to the randomisation in the trial and found that patients prescribed digoxin pre-trial were more likely to have advanced HF, compared to those who were not [154].

In a recent meta-analysis of eight studies, Hood *et al*. [155] showed that digoxin reduced the rates of hospitalisation and clinical deterioration in patients with HF with or without atrial fibrillation. It, similar to the DIG trial, did not show an effect on mortality.

#### 8.1.2 Digoxin in HFmrEF/HFpEF 

The DIG ancillary trial recruited patients with LVEF >45% with the same serum creatinine cut-off. This trial did not show a difference in either mortality, nor all-cause hospitalisation [156]. Observational studies have similarly shown either no effect, or increased mortality and hospitalisation in patients treated with digoxin, compared to those who were not [157, 158]. The increased mortality rate and hospitalisation in some observation studies may, similar to HFrEF, be due to prescription bias as digoxin is usually prescribed to patients with more advanced HF.

The pivotal DIG trial was conducted more than 20 years ago. There are RCT’s currently being conducted, investigating the efficacy of digoxin in the current age of widespread use of beta-blockers and various other HF drugs that were not in use at the time of the DIG trial [159, 160].

### 8.2 Ivabradine 

Heart rate reduction using beta blockers has been shown to improve cardiovascular outcomes and mortality in patients with HFrEF [161]. Furthermore, the I-PRESERVE trial identified resting heart rate as an independent predictor of adverse clinical outcomes [72]. Thus, medications to lower heart rate are desirable in HF, however, beta-blockers have limitations due to their effect on other body systems, and thus, are limited in certain patient groups such as those with asthma. Ivabradine is a selective inhibitor of the sino-atrial ‘funny’ pacemaker channel, and thus lowers the heart rate very specifically [162].

#### 8.2.1 Ivabradine in HFrEF 

The Morbidity-Mortality Evaluation of the If Inhibitor Ivabradine in Patients With Coronary Artery Disease and Left Ventricular Dysfunction (BEAUTIFUL) trial (2008) recruited 10,917 patients with HFrEF and stable coronary artery disease, and randomised participants to receive either ivabradine or placebo [163]. The trial excluded patients with severe renal disease. This trial demonstrated that ivabradine reduced heart rate by 6 beats per minute compared to placebo at 12 months. At a median follow-up of 19 months (Interquartile range, IQR 16–24), ivabradine did not reduce the rates of hospitalisations or mortality. However, curiously, there was an effect in a subgroup of patients who had a resting heart rate of >70 bpm in reducing admission to hospital for fatal or non-fatal myocardial infarction (HR 0.64; 95% CI 0.4–0.83; *p* = 0.001) and for coronary revascularization (HR 0.70; 95% CI 0.52–0.93; *p* = 0.016). In addition, since trial patients were able to use concomitant beta-blockers along with ivabradine as the study drug, this trial showed that the concomitant prescription of ivabradine with beta-blockers was safe. Adverse events were similar across ivabradine and the placebo group (36.12 Patient-years vs 34.73 Patient-years, *p* = 0.02).

The Systolic Heart Failure Treatment With the If Inhibitor Ivabradine Trial (SHIFT) trial randomised 6558 patients with stable HFrEF (LVEF <35%) who were established on a stable dose of beta-blocker, to either ivabradine or placebo, and demonstrated a reduction in death due to HF (HR 0⋅74; 95% CI 0.58–0.94; *p* = 0.014) and HF hospitalisation (HR 0.74; 95% CI 0.66–0.83; *p* < 0*.*0001) [164]. In a subgroup analysis, a significant treatment effect for the composite outcome of mortality or hospitalisation due to HF was only found for patients with a resting heart rate of >77 bpm. SHIFT excluded patients with serum creatinine of >220 umol/L and reported a similar eGFR across the ivabradine and placebo group (74.6 ± 22.9 vs 74.8 ± 23.1 mL/min/1.73 $m^{2}$). In a secondary analysis of the SHIFT trial, Voors *et al*. [165] showed no differences in renal function changes over 24 months of follow up, between ivabradine and placebo (*p* = 0.36).

There is currently little evidence regarding the efficacy of ivabradine in patients with CKD Stage G4-5 or on renal replacement therapy. However, there are a few case reports suggesting patients with HFrEF suffering from intra-hemodialytic hypotension may benefit from ivabradine over beta-blocker [166, 167]. They suggest ivabradine may allow for a negative chronotropic effect without a negative inotropic effect, therefore allow a more stable blood pressure during hemodialysis treatment.

#### 8.2.2 Ivabradine in HFpEF 

The evidence for ivabradine in patients with HFpEF is conflicting. Cacciapuoti *et al*. [168] showed that 25 patients with HFpEF had an increased LVEF after three months of treatment with ivabradine (48.0 ± 0.20 vs 51.0 ± 0.12, *p <* 0.05). Tanaka *et al*. [169] conducted a similar study in 16 patients, showing no increase in LVEF (64.2 ± 7.7 vs 64.2 ± 6.8, *p* = 0.66) after three months of treatment with ivabradine. There were also no differences in mitral inflow E and mitral e’ annular velocities (E/e’; 12.1 ± 4.4 vs 13.6 ± 4.1, *p* = 0.16). In the The Preserved Left Ventricular Ejection Fraction Chronic Heart Failure with Ivabradine Study (EDIFY) trial [170], ivabradine did not improve echo-Doppler E/e’ ratio (Between-group estimate 1.4, 90% CI 0.3–2.5, *p* = 0.135), distance walked on a 6 minute walking test (Between-group estimate –3.8, 90% CI –19.1–11.6, *p* = 0.882), nor plasma NT-proBNP concentration (ratio 1.01, 90% confidence interval –0.86 to 1.19; *p* = 0.882) in patients with HFpEF after 8 months of treatment.

### 8.3 Vericiguat

Vericiguat is a soluble guanylate cyclase stimulator that helps potentiate nitric oxide action on the smooth muscle cells [171]. Patients with HF suffer from endothelial dysfunction which reduces the bioavailability of nitric oxide. Vericiguat is thought to produce a more physiological effect of increasing nitric oxide compared to isosorbide dinitrate (ISDN) and hydralazine, thereby reducing the common side effects of hypotension and syncope [172].

#### 8.3.1 Vericiguat in HFrEF 

The Vericiguat Global Study in Subjects With Heart Failure With Reduced Ejection Fraction (VICTORIA) trial recruited HF patients with a LVEF of <40%. The trial capped the number of patients recruited with eGFR of 15–30 mL/min/1.73 $m^{2}$ to 15% of trial total population [173]. The trial had a mean eGFR of 61 mL/min/1.73 $m^{2}$. This trial showed that treatment with vericiguat for a median of 10.8 months reduced the composite outcome of death from any cause or hospitalisation for HF (HR 0.90; 95% CI 0.83–0.98; *p* = 0.02). Symptomatic hypotension (Vericiguat 9.1% vs Placebo 7.9%, *p* = 0.12) and syncope (Vericiguat 4.0% vs Placebo 3.5%, *p* = 0.30) occurred at similar rates across the treatment and placebo groups. In a secondary analysis of the VICTORIA trial, Voors *et al*. [174] showed that the trajectories eGFR and serum creatinine across 48 weeks of the trial were similar between Vericiguat and placebo group (*p* = 0.50 and *p* = 0.18 respectively). The beneficial effect of vericiguat was also shown to be consistent across the range of eGFR within the VICTORIA trial (Interaction *p* = 0.48). However, patients with worsening renal function during the trial (increase in creatinine ≥0.3 mg/dL from baseline to week 16) were found to have higher risk of HF admission or all-cause mortality (HR 1.24; 95% CI 1.08–1.43; *p* = 0.002) after adjusting for clinical factors such as NYHA classification.

#### 8.3.2 Vericiguat in HFpEF

Soluble guanylate cyclase stimulator in heart failure with preserved ejection fraction (SOCRATES-PRESERVED) is a Phase 2b dose-finding trial of vericiguat in HFpEF [175]. Pieske *et al*. [175]. showed that vericiguat is well tolerated, with adverse events similar between vericiguat and placebo arm of the trial during 12 weeks of follow up (Vericiguat 10 mg arm 79.8% vs placebo 73.1%). Patient reported outcomes, measured by Kansas City Cardiomyopathy Questionnaire Clinical Score (KCCQ), was positively associated with vericiguat dose (Slope (SD) 0.92 (0.29), *p* = 0.0017). However, there were no changes in primary endpoints NT-proBNP (0.038 0.782 log(pg/mL) vs –0.098 0.778 log(pg/mL), *p* = 0.20) or left atrial volume (–1.7 ± 12.8 vs –3.4 ± 12.7, *p* = 0.37). This trial excluded patients with eGFR < 30 mL/min/1.73 $m^{2}$ and had a mean eGFR of 54.8 (20.3) across its study sample [176]. In a secondary analysis, Filippatos showed clinically important improvements in health status was associated with vericiguat as assessed by both KCCQ and EuroQol-5 dimension quality of life questionnaire (EQ-5D) [177].

In another Phase 2b trial VITALITY-HFpEF, Armstrong *et al*. [178], showed after 24-week up-titration with max-dose vericiguat 15 mg/day or 10 mg/day compared with placebo, there were no improvements with the physical limitation score of KCCQ (Mean different –1.5; 95% CI –5.5–2.5; *p* = 0.46) (–0.5; 95% CI –4.6–3.5; *p* = 0.80). There was also no difference in 6-minute walking distance between 15 mg/day with placebo (Mean difference –5.5; 95% CI –19.7–8.8; *p* = 0.45), nor with 10mg/day and placebo (mean difference –1.8; 95% –16.2–12.6; *p* = 0.81). This trial similarly excluded patients with eGFR <30 mL/min/1.73 $m^{2}$ [179]. This trial had 147 (55.7%), 123 (46.8%), and 155 (59.2%) patients with eGFR ≤60 mL/min/1.73 $m^{2}$ in Vericiguat 15 mg/day arm, Vericiguat 10 mg/day arm, and Placebo arm, respectively. There is a need for more evidence with vericiguat usage in patients with HFpEF.

### 8.4 Isosorbide Dinitrate & Hydralazine

The first trial of isosorbide dinitrate (ISDN) with hydralazine was conducted in the 1980s – the Vasodilator Heart Failure Trial (V-HeFT I) trial [180]. ISDN was originally thought to act as a nitric oxide donor to increase the bioavailability of nitric oxide, however recent evidence has shown it may have a more complex pathway involving several enzymes within the body [181]. Meanwhile hydralazine is prescribed to reduce the risk of the body from developing a tolerance to ISDN.

In 1986, V-HeFT I reported their results, showing treatment with ISDN + Hydralazine reduced mortality across a follow up period of about 2 years compared to treatment with Prazosin or with placebo [180]. This was superseded by the V-HeFT II study published in 1991, where they found enalapril was more effective than hydralazine-ISDN arm [56]. However, curiously, in a secondary analysis of the V-HeFT I & II datasets, Carson *et al*. [182] showed that the mortality benefit of enalapril and hydralazine-ISDN was not statistically significant (*p* = 0.67).

The The African American Heart Failure Trial (A-HeFT) trial sought to explore this difference by recruiting patients who self-identify as black (defined as of African descent) with LVEF <35% or a dilated left ventricle with a LVEF of <45% [183]. This trial showed significantly higher mortality rates in patients in the placebo group compared to the hydralazine and ISDN group (10.2% vs 6.2%, *p* = 0.02). It also showed reduced rate of hospitalisation for HF (16.4% vs 22.4%, *p* = 0.0001) and an improved quality of life as measured by the Minnesota Living with HF questionnaire where lower scores mean higher quality of life (mean change in score –5.6 ± 20.6 vs –2.7 ± 21.2, *p* = 0.02). This trial was terminated early due to the difference in mortality between the treatment and placebo arm of the trial, the mean follow-up duration was 10 months (range 0–18 months).

In a RCT with patients with HFpEF, Zamani *et al*. [184] showed that ISDN, with or without hydralazine, did not reduce wave reflections, left ventricular hypertrophy, nor myocardial fibrosis compared to placebo. Hydralazine with ISDN may not have a role in treating HFpEF.

Genetic Risk Assessment and HF, a substudy of A-HeFT, is an exploratory study looking at whether there is a more specific genetic identifier for the reason why patients who identify as black or of African descent would respond to hydralazine with ISDN more than patients who identify as white [185]. Genomic Response Analysis of Enhanced Heart Failure Therapy in African Americans (GRAHF2) may be able to confirm these hypotheses and identify the genes responsible for this difference in response to hydralazine and ISDN [186].

## 9. Devices

### 9.1 ICD 

Currently, NICE, ESC and the American Heart Association (AHA) all recommend that patients with a high risk of sudden cardiac death are treated with an implantable cardioverter-defibrillator (ICD) [187, 188, 189]. This includes patients with a prolonged QRS interval, or patients who have had a previous serious ventricular arrhythmia with no treatable cause. It is recommended that cardiac resynchronization therapy (CRT) (with or without a defibrillator) or a pacemaker is offered to patients with a prolonged QRS interval, with a LVEF ≤35%, and NYHA classification of II–IV [188].

#### 9.1.1 ICD in HFrEF 

In the Multicenter Automatic Defibrillator Implantation Trial II (MADIT II) trial, 1232 patients with a previous myocardial infarction and LVEF <30% were randomised to receive either an ICD or standard medical therapy [190]. There was a reduced risk of death from any cause in the ICD group compared to the standard medical therapy group (HR 0.69; 95% CI 0.51–0.93; *p* = 0.016) over a follow up period of 20 months (range 6 days to 53 months). The trial excluded patients with serum creatinine >3 mg/dL (265 µmol/L). However, approximately 387 patients (31.6%) had CKD Stage G3a. A subgroup analysis revealed that ICD efficacy declined with worsening renal function, and there was no benefit found for patients with eGFR <35 mL/min/1.73 $m^{2}$ (HR 1.09; 95% 0.49–2.43; *p* = 0.84) [191]. eGFR was higher in the ICD group compared to the conventional group (70.3 ± 24.9 vs 66.5 ± 20.8, *p* = 0.004) [191]. Kaplan-Meier estimates of all-cause mortality at 2 years showed mortality rates increased across decreasing eGFR categories in the ICD and standard medical therapy group (ICD group 11%, 20%, and 39%, *p* < 0.001, standard medical therapy group 16%, 31%, and 37%, *p <*0.001, for eGFR categories of ≥60, 35–59, and <35 mL/min/1.73 $m^{2}$ respectively).

In Sudden Cardiac Death in Heart Failure Trial (SCD-HeFT) trial, patients with LVEF <35% were randomised to receive either an ICD or amiodarone, plus standard medical therapy [192]. This trial confirmed ICD group had a reduced risk of death compared to placebo and standard medical therapy group (HR 0.77, 97.5% CI 0.62–0.96, *p* = 0.007) at a median follow up of 45.5 months. Of the participants who completed this trial, 51.7% had an eGFR of <60 mL/min/1.73 $m^{2}$, and 10.3% had an eGFR of <30 mL/min/1.73 $m^{2}$ [193].

In a meta-analysis of three ICD trials, including 2867 patients, Pun *et al*. [193] showed that there was a significant interaction between eGFR and the benefit of ICD to all-cause mortality (posterior probability *p <* 0.001). It also showed that there was no statistically significant all-cause mortality benefit obtained with ICD’s in patients with eGFR <60 mL/min/1.73 $m^{2}$.

#### 9.1.2 ICD in HFmrEF/HFpEF 

In the ICD2 trial, patients with LVEF ≥35% and on haemodialysis were recruited to receive an ICD or standard medical therapy [194]. ICD did not reduce the rate of all-cause mortality when compared against standard medical therapy (HR 1.02; 95% CI 0.69–1.52; *p* = 0.92). However, there may be a role for ICD therapy in secondary prevention in this patient group. Herzog *et al*. [195] showed a reduction in overall risk of death in dialysis patients who had been hospitalized for cardiac arrest that received ICD within 30 days of admission compared to those who did not (HR 0.58; 95% CI 0.50–0.66; *p* < 0.0001).

Subcutaneous ICDs may be a suitable device to use in patients with CKD or haemodialysis as it avoids the vascular issues in transvenous ICDs. Two observational studies have shown similar procedural outcomes and inappropriate shocks in haemodialysis and non-haemodialysis patients [187, 196].

### 9.2 CRT 

Various pivotal clinical trials have demonstrated clear benefits of CRT in HFrEF in terms of symptoms, quality of life, hospitalisation, and risk of death [197, 198, 199, 200]. Cardiac-Resynchronization Therapy for Mild-to-Moderate Heart Failure (RAFT-HF) had 43% of patients with CKD stage G3 and found no significant interaction between baseline renal function and the treatment effect of CRT [199]. Furthermore, in a secondary analysis of Multicenter InSync. Randomized Clinical Evaluation (MIRACLE), Boerrigter *et al*. [201] showed that patients with CKD stage G3 who received CRT had improved eGFR compared to controls.

In a secondary analysis of Multicenter Automatic Defibrillator Implantation Trial – Cardiac Resynchronization Therapy (MADIT-CRT) & Ranolazine in High-Risk Patients with Implanted Cardioverter Defibrillator (RAID) trial, Goldenberg *et al*. [202] showed there is a lower incidence of Ventricular tachycardia (VT)/Ventricular fibrillation (VF) in patients with CKD Stage G3b-5 compared to patients with CKD Stage G1-3a (HR 0.56; 95% CI 0.33–0.94; *p* = 0.03) who were enrolled in either trial. There was a higher risk of death without any VT/VF among patients with CKD Stage G3b-5 compared to CKD Stage G1-3a (HR 4.63; 95% CI 2.46–8.72; *p* = 0.01). This suggests the benefit of ICD may be attenuated in CRT recipients with renal impairment due to the reduced incidence of arrhythmias and higher risk of death without arrhythmia.

There has been some interesting development in wireless CRT and ICD, for example, Boveda *et al*. [203] showed leadless pacemakers had lower reintervention and complication rates compared to transvenous pacemakers in high risk patients including patients with CKD stage G4-5. These devices may offer advantages by avoiding difficulties regarding vascular access, especially in patients on hemodialysis. Micra from Medtronic has offered.

## 10. Revascularisation 

Revascularisation in patients with HF from ischaemic cardiomyopathy, and patients with ischaemic heart disease and CKD has been explored previously in RCT’s. Revascularization for Ischemic Ventricular Dysfunction (REVIVED-BCIS2) [204] recruited patients with LVEF <35%, with extensive coronary artery disease. This study excluded patients with eGFR <25 mL/min/1.73 $m^{2}$ but included patients on dialysis. This study showed that over a median time of 41 months, the composite outcome of death from any cause or hospitalisation for HF was similar across patients who underwent percutaneous coronary intervention (PCI) or just optimal medical therapy (HR 0.99, 95% CI 0.78–1.27, *p *= 0.96).

The Surgical Treatment for Ischemic Heart Failure (STICH) trial [205] recruited patients with LVEF <35% with coronary artery disease amenable to Coronary Artery Bypass Graft (CABG). These patients were subsequently randomized to receive either CABG or just medical therapy. STICH found that the addition of CABG did not statistically significantly reduce the number of cardiovascular deaths (HR 0.83, 95% CI 0.68–1.03, *p *= 0.09).

The International Study of Comparative Health Effectiveness With Medical and Invasive Approaches (ISCHAEMIA)-CKD trial [206] recruited patients with eGFR <30 mL/min/1.73 $m^{2}$ or end-stage renal disease on dialysis. However, this study excluded patients with heart failure of NYHA classification 3–4 and patients with LVEF <35%. This study compared revascularization (PCI or CABG) against optimal medical therapy. This showed that the initial invasive strategy increased the incidence of stroke (HR 3.76, 95% CI 1.52–9.32, *p *= 0.004) and a higher incidence of death or initiation of dialysis (HR 1.48, 95% CI 1.04–2.11, *p *= 0.03).

## 11. Iron & Anaemia 

There is an intricate relationship between HF, CKD, and iron deficiency, along with its associated anaemia [207]. The iron deficiency status in HF and CKD is likely associated with patients low grade inflammatory status, and overstimulation of the sympathetic nervous system and renin-angiotensin system.

IV iron therapy has been shown to be superior to oral iron therapy in patients with HF and CKD [208]. This may be due to poor intestinal absorption of iron in patients with HF and CKD. However, IV iron is more expensive and logistically more challenging, and thus, depending on patient preferences and individual case specifics, there may still be a role for oral iron therapy in this cohort.

IV iron has been shown to improve quality of life, relieve symptoms of HF, and reduce the risk of hospitalisation in a series of RCT’s, including Ferric carboxymaltose Assessment in patients with IRon deficiency and chronic Heart Failure (FAIR-HF) [209], Ferric CarboxymaltOse evaluatioN on perFormance in patients with IRon deficiency in coMbination with chronic Heart Failure (CONFIRM-HF) [210], Effect of Ferric Carboxymaltose on Exercise Capacity in Patients With Chronic Heart Failure and Iron Deficiency (EFFECT-HF) [211], and Study to Compare Ferric Carboxymaltose With Placebo in Patients With Acute Heart Failure and Iron Deficiency (AFFIRM-AHF) [212]. In a meta-analysis of these studies, Osman *et al*. [213] demonstrated that IV iron therapy reduced hospitalisation for HF (pooled RR 0.69; 95% CI 0.61–0.78; *p* = 0.043) after a mean follow up of 31 ± 14 weeks. However, there was no difference between IV iron therapy and standard of care in all-cause mortality (pooled RR 0.67; 95% CI 0.36–1.23; *p* = 0.37). More recently, the Ferric Carboxymaltose in Heart Failure With Iron Deficiency (HEART-FID) study investigating IV iron in 3065 patients with HFrEF and iron deficiency, failed to reach significance for its primary endpoint (composite of all-cause mortality, HF hospitalisation or change in 6-minute walking distance), *p* = 0.19 [214]. However, this large study did demonstrate safety of IV iron and demonstrated a trend favouring IV iron in each of the components of the primary outcome. In another recent meta-analysis, Anker *et al*. [215] showed a reduction in composite outcome of total cardiovascular hospitalisation and CV death (pooled RR 0.86; 95% CI 0.75–0.98; *p* = 0.029). Since most RCT’s did not exclude patients with CKD (AFFIRM-AHF had 40% of patients who had CKD Stage G3 or lower), these results likely extend to patients with renal impairment.

There is currently little available evidence for iron therapy in patients with HFpEF. The FAIR-HFpEF will hopefully provide answers to the role of IV iron in HFpEF [216].

Currently, clinical trials have demonstrated that Hypoxia-Inducible Factor-Prolyl Hydroxylase Domain Inhibitors such as Roxadustat are effective and safe, and are being discussed with patients with CKD who are established on dialysis [217]. However, there is currently no evidence for their role in HF, with or without CKD. In the future, it is hoped that Iso *et al*. [218] will be able to answer this question with a RCT in patients with HF and CKD.

## 12. Frailty

Frailty is a prevalent condition, defined by an increased vulnerability to stressors due to cumulative deficiencies in several physiological domains [219]. Frailty is very common in both patients with HF and patients with CKD [220]. Frailty can be defined using several tools; the most utilised of which include the ‘Clinical Frailty Scale’ and the ‘Modified Frailty Phenotype’, although neither score have been validated specifically in patients with HF [221].

Polypharmacy is a risk factor for frailty, and consequently, patients with HF and frailty may be less likely to be prescribed the optimal evidence-based medications for HF [219]. However, separate post-hoc analysis of some of the above described RCT’s consistently demonstrate that frailty is common, patients living with frailty are most at risk of adverse outcomes and that frail patients benefit most from these medications [222, 223, 224, 225].

Furthermore, in an analysis of the DELIVER trial, eGFR was significantly lower in the most frail vs least frail group (52.1 ± 17.4 vs 68.7 ± 18.0) [223].

It is imperative to take a holistic and individualised approach to the management of frailty. As recommended above, it is important to monitor clinical parameters of concern in patients after commencing any of the evidence-based therapies, e.g., blood pressure in antihypertensive medications, and to remain vigilant for when the burden of medication may outweigh its potential benefit in individuals. Furthermore, the management of frailty should be holistic, and involve not only medications, but also nutritional, cognitive and physical interventions [219]. Crucially, the presence of frailty alone should not impede the prescription of evidence-based therapeutics.

## 13. Discussion

There has been remarkable progress in recent years in this area prompting an early focused update of the 2021 ESC HF guidelines by the task force in 2023. Based on the EMPEROR-Preserved [18], DELIVER [19], and EMPA-KIDNEY [140] trials, SGLT2i’s were recommended for all patients regardless of LVEF, CKD or diabetic status. The evidence provided by IRONMAN (Effectiveness of IV Iron Treatment Versus Standard Care in Patients with HF and Iron Deficiency) [226] and AFFIRM-AHF [212] trials supports the use of IV Iron in patients with HFrEF to improve symptom control and hence quality of life. Finerenone in Reducing Kidney Failure and Disease Progression in Diabetic Kidney Disease (FIDELIO-DKD) [227] and FIGARO-DKD [228] have provided evidence on safety and efficacy of non-steroidal MRA use in patients with a range of CKD severity and type 2 diabetes and concluded that Finerenone lowered the risk of CKD progression and cardiovascular events in this high-risk population.

Prevention of HF remains an important area of clinical concern and research. Patients at high risk of developing CKD and HF, especially those with type 2 diabetes, should be monitored regularly to ensure steps are taken in a timely fashion to prevent cardiorenal complications. American Diabetes Association (ADA) recommends yearly evaluation of all patients with type 2 diabetes for renal function (eGFR) and urinary albumin levels, with use of SGLT2i, RAASi (ACEi, ARB, ARNI) and MRA as tolerated by patients, using a patient tailored approach [229].

Whilst temporary discontinuation of medication such as RAASi may be appropriate acutely (e.g., for acute kidney injury on a background of CKD and/or acute decompensation of chronic HF), the results of the STOP-ACEi trial has reassured us that in case of progressive and/or advanced CKD, stopping RAASi does not affect the long-term rate of decline in renal function [83].

**Fig. 2.**
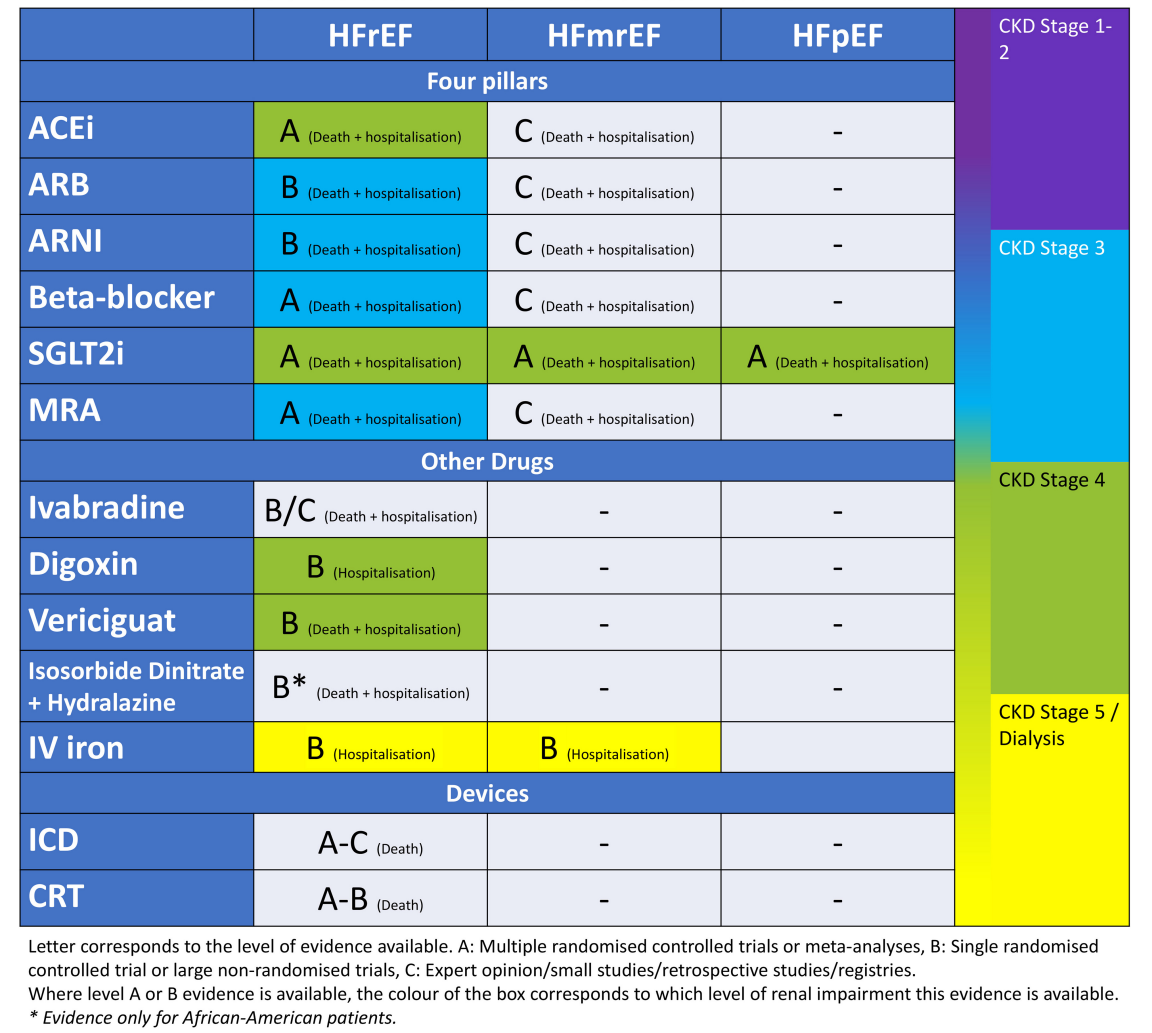
**A summary diagram of the available evidence for interventions to reduce risk of HF hospitalisation and death in patients with HF**. ACEi, angiotensin converting enzyme inhibitor; ARB, aldosterone receptor blocker; ARNI, angiotensin receptor-neprilysin inhibitor; SGLT2i, sodium-glucose cotransporter-2 inhibitor; MRA, mineralocorticoid receptor antagonist; IV, intravenous; ICD, implantable cardioverter defibrillator; CRT, cardiac resynchronisation therapy; HF, heart failure; HFrEF, heart failure with reduced ejection fraction; HFmrEF, heart failure with mildly reduced ejection fraction; HFpEF, heart failure with preserved ejection fraction.

Chronic HF in context of CKD remains a challenging scenario for clinicians to manage, which is usually complicated by frailty, multimorbidity and polypharmacy. It is important to ensure that these patients are assessed carefully and commenced on the recommended HF treatment as tolerated: the four pillars of HF treatment (beta-blockers, RAASi [ACEi, ARB, ARNI], MRA and SGLT2i), diuretics as appropriate to ensure adequate decongestion, iron therapy to improve symptom control, and use of device therapy as indicated (summarised in Fig. 2), whilst being monitored closely for worsening renal function and hyperkalemia. Patients should be educated regarding the sick day rules to reduce likelihood of worsening renal function and hyperkalaemia. The treatment should be tailored to individual patient needs and hence management in specialised cardio-renal clinics with a multi-disciplinary team approach has been recommended to provide a more holistic care to this complex patient group [230, 231, 232].

## Abbreviations

ACEi, angiotensin coverting enzyme inhibitors; ARB, angiotensin II receptor blocker; ARNI, angiotensin receptor neprilysin inhibitor; BD, twice per day; BNP, B-type natriuretic peptide; CKD, chronic kidney disease; CI, confidence interval; CRT, cardiac resynchronization therapy; CVS, cardiovascular; eGFR, estimated glomerular filtration rate; ESC, european society of cardiology; HR, hazard ratio; HF, heart failure; HFmrEF, heart failure with mildly reduced ejection fraction; HFpEF, heart failure with preserved ejection fraction; HFrEF, heart failure with reduced ejection fraction; ICD, implantable cardioverter-defibrillator; IQR, interquartile range; IV, intravenous; KCCQ, kansas city cardiomyopathy questionnaire; ISDN, isosorbide dinitrate; LVEF, left ventricular ejection fraction; MRA, mineralocorticoid receptor antagonist; NT-proBNP, N-terminal pro-B-type natriuretic peptide; NYHA, New York Heart Association; OD, once per day; OR, odds ratio; RAAS, renin-angiotensin-aldosterone system; RR, relative risk; RCT, randomised controlled trial; SGLT2i, sodium-glucose co-transporter-2 inhibitor; TDS, three times per day.

## Appendix

**Sick Day Rules **

STOP SGLT2i if feeling unwell for at least 24–48 hours, or until recovery to normal and eating drinking normally.

Resume SGLT2i as directed once recovered.

Seek medical attention if still feeling unwell >48 hours.

**Other things to remember **

Chronic kidney disease: Initiate if eGFR >20 mL/min/1.73 $m^{2}$. SGLT2i’s can be continued at lower eGFR levels once initiated. Optimise volume status before commencement.

Major surgery: Consider stopping SGLT2i three days before the operation.

Older adults: SGLT2i use considered safe to use in older adults. Monitor for decreased intravascular volume and hypotension.

Pregnancy and breast feeding: Contraindicated in pregnancy and not advised during breastfeeding.
